# Interventions targeting healthcare providers to optimise use of caesarean section: a qualitative comparative analysis to identify important intervention features

**DOI:** 10.1186/s12913-022-08783-9

**Published:** 2022-12-14

**Authors:** Rana Islamiah Zahroh, Dylan Kneale, Katy Sutcliffe, Martha Vazquez Corona, Newton Opiyo, Caroline S. E. Homer, Ana Pilar Betrán, Meghan A. Bohren

**Affiliations:** 1 Gender and Women’s Health Unit, Centre for Health Equity, School of Population and Global Health, The University of Melbourne, Melbourne, VIC Australia; 2 EPPI-Centre, UCL Social Research Institute, University College London, London, UK; 3 UNDP/UNFPA/UNICEF/WHO/World Bank Special Programme of Research, Development and Research Training in Human Reproduction (HRP), Department of Sexual and Reproductive Health and Research, World Health Organization, Geneva, Switzerland; 4 Maternal, Child, and Adolescent Health Program, Burnet Institute, Melbourne, VIC Australia

**Keywords:** Maternal health, Caesarean section, Qualitative comparative analysis, Complex intervention, Intervention implementation

## Abstract

**Background:**

Rapid increases in caesarean section (CS) rates have been observed globally; however, CS rates exceeding 15% at a population-level have limited benefits for women and babies. Many interventions targeting healthcare providers have been developed to optimise use of CS, typically aiming to improve and monitor clinical decision-making. However, interventions are often complex, and effectiveness is varied. Understanding intervention and implementation features that likely lead to optimised CS use is important to optimise benefits. The aim of this study was to identify important components that lead to successful interventions to optimise CS, focusing on interventions targeting healthcare providers.

**Methods:**

We used Qualitative Comparative Analysis (QCA) to identify if certain combination of important intervention features (e.g. type of intervention, contextual characteristics, and how the intervention was delivered) are associated with a successful intervention as reflected in a reduction of CS. We included 21 intervention studies targeting healthcare providers to reduce CS, comprising of 34 papers reporting on these interventions. To develop potential theories driving intervention success, we used existing published qualitative evidence syntheses on healthcare providers’ perspectives and experiences of interventions targeted at them to reduce CS.

**Results:**

We identified five important components that trigger successful interventions targeting healthcare providers: 1) training to improve providers’ knowledge and skills, 2) active dissemination of CS indications, 3) actionable recommendations, 4) multidisciplinary collaboration, and 5) providers’ willingness to change. Importantly, when one or more of these components are absent, dictated nature of intervention, where providers are enforced to adhere to the intervention, is needed to prompt successful interventions. Unsuccessful interventions were characterised by the absence of these components.

**Conclusion:**

We identified five important intervention components and combinations of intervention components which can lead to successful interventions targeting healthcare providers to optimise CS use. Health facility managers, researchers, and policy-makers aiming to improve providers’ clinical decision making and reduce CS may consider including the identified components to optimise benefits.

**Supplementary Information:**

The online version contains supplementary material available at 10.1186/s12913-022-08783-9.

## Introduction

Rapid increases in caesarean section (CS) rates have been observed globally in recent years [1–4]; however, CS rates exceeding 15% at a population-level have limited benefits for women and babies [1, 5]. Globally, CS rates have increased from around 5.0% in 1990 to 21.1% in 2018, and projected to reach 28.5% by 2030 [3]. CS is a life-saving surgical procedure for women and babies when vaginal birth is not possible. Despite the life-saving benefits, women undergoing CS are at risk of haemorrhage, anaesthetic complications, obstetric shock, renal failure, puerperal infection, and complications in subsequent pregnancies [6–9]. Similarly, babies born through CS have increased risk of respiratory problems, hypoglycaemia, allergies and altered immunity [10–12]. Therefore, optimising use of CS is critical to maximise benefits and avoid unnecessary risks for women and babies.

While CS should be done based on medical indications, non-clinical factors have been increasingly driving high CS rates. Some women may prefer to have CS over vaginal birth due to fears undergoing vaginal birth, negative previous birth experiences, prioritising the baby’s life, choosing an “auspicious” day of birth, perceptions that CS is safer, quick and painless, and the financial ability to choose “better” healthcare services [13–16]. Similarly, healthcare providers may prefer CS due to the perception that it is a safer option than vaginal birth, the convenience of scheduled CS compared to the unpredictability of vaginal birth, a preference to play safe instead of taking risks in being blamed if complications occur during vaginal birth, and desire to respect women’s autonomy when they opt for CS over vaginal birth [13, 14, 17]. Exacerbating this further, health systems also play a role in encouraging healthcare providers and women to have CS. This can be through financial structures in which higher financial incentives are given for CS compared to vaginal birth, logistical needs which includes inadequacy of labour rooms, expectations around time in using existing labour facilities, unequal power relationships between providers, and culture of medicalisation of birth [17, 18].

Globally, interventions targeting healthcare providers to change their behaviours around CS have been tested. A Cochrane intervention review identified 16 intervention studies targeting healthcare providers and systems, typically focusing on implementation of audit and feedback of CS data combined with either implementation of clinical guidelines and protocols, mandatory secondary opinion for CS decision-making, or working with local opinion leaders to influence change [19–21]. Audit and feedback can help healthcare providers to assess the distribution of CS across different groups of women, and identify areas for appropriate increases or decreases in these groups based on obstetric characteristics [22]. Hospital policies of mandatory secondary opinion at the time of decision-making may influence healthcare provider attitudes regarding indication for CS [23]. Similarly, local opinion leaders can change organisational culture by modelling appropriate behaviour to their colleagues [24].

Across contexts and studies, the effectiveness of interventions targeting healthcare providers in reducing CS has had mixed results [19]. Contextual characteristics of the interventions (i.e. participant characteristics, intensity of exposure to the intervention, implementation method, type of health facility) may impact implementation and thus influence effectiveness of the intervention. Therefore, understanding which intervention components, and in which contexts, are important to the success of interventions is imperative to optimise benefits of CS. The aim of this study was to identify important intervention components that lead to successful non-clinical interventions targeting healthcare providers to optimise the use of CS.

## Methods

### Qualitative comparative analysis (QCA)

Utilising QCA, we re-analysed evidence from existing systematic reviews using new analytical frameworks to explore the heterogeneity in effects and ascertain why some studies appear to be effective while others not. QCA is often conceptualised as a “bridge between qualitative and quantitative methodologies” based on its data, process and theoretical standpoint [25]. QCA is an evidence synthesis method that allows researchers to explore different commonly occurring characteristics of complex interventions [26]. These properties include recognition that different pathways may lead to the same outcome (equifinality), and that intervention components may only be activated to have an influence on the outcome in the presence of other components (conjunctural causation) [26].

QCA facilitates comparisons of intervention components – referred as “conditions” in QCA – present in successful (effective interventions) and unsuccessful (ineffective interventions) “cases” [26, 27]. This is done using a scoring system based on “set membership”. In this scoring system, all potential conditions and outcomes are coded based on the extent to which they are present or absent to form set membership [26, 27]. There are two types of scoring systems in QCA, which are crisp set QCA (csQCA) and fuzzy set QCA (fsQCA) [26, 27]. In csQCA, conditions and outcomes are coded to binary values either to 0 (“fully out” of set membership) or 1 (“fully in” set membership) [26, 27]. By “fully out”, we mean the condition is entirely absent, while “fully in” means that the condition is entirely present. In fsQCA, the conditions and outcomes can be coded in ordinal values between 0 to 1 [26, 27]. In our study, we adopted both csQCA and fsQCA to code our data, as some data have explicit binary options (e.g. yes, no), while others are more nuanced (e.g. adherence or participant satisfaction). However, our final analysis only includes conditions using csQCA scoring system due to the explicit binary nature of the data included in the final solutions.

QCA is based on set-theory in which two types of relationships are explored: necessary and sufficient [25, 26]. When all successful interventions share the same exact condition(s), this condition(s) is deemed “necessary” to trigger successful interventions [25, 26]. Necessary condition must be present to prompt successful interventions, yet necessary condition alone do not provide sufficient cause for successful intervention [25, 26]. However, when all instances of a particular condition(s) is associated with successful interventions, this condition(s) is “sufficient” to trigger successful interventions [25, 26], although other pathways towards a successful intervention may also exist. In our QCA, we were interested in exploring “sufficiency”, as our logic model (Additional File 1) highlighted that there are multiple pathways to optimise CS, suggesting that it would be unlikely for all successful interventions to share the same conditions. The degree of sufficiency was calculated using consistency scores, which measure the frequency in which conditions are present when the desired outcome is achieved [26, 27]. We conducted the QCA using R programming software with QCA package developed by Thiem and Duşa and QCA with R guidebook [27]. QCA was conducted in six stages based on Thomas et al. (2014) [26] and explained below.

### Data sources, case selection, and defining outcomes

#### Developing a logic model

We developed a logic model to guide our understanding about the different pathways and intervention components potentially leading to successful implementation (Additional File 1), which was based on existing qualitative evidence syntheses and quantitative systematic reviews [18, 19, 25, 28–30]. With the logic model, we worked backwards to understand what inputs are needed to achieve our desired outcome, that is reduced CS rates in what it has been conceptualized as low-risk women (e.g. women with term, singleton, cephalic pregnancies without previous CS, who are typically represented by the Robson groups 1–4 [31]). The logic model was used to guide the analysis.

#### Identifying data sources and selecting cases

In 2018, World Health Organization (WHO) issued global guidance on non-clinical interventions to reduce unnecessary CS, with interventions designed to target three different stakeholders: women, healthcare providers, and health systems [32]. As part of the guideline development, a series of systematic reviews about CS interventions were conducted: 1) a Cochrane intervention review of effectiveness by Chen et al. [19], and 2) three qualitative evidence syntheses exploring key stakeholders (women and communities, health professionals, and health systems) perspectives and experiences of CS interventions by Kingdon et al. [18, 28, 29]. Following this, Opiyo and colleagues published a scoping review of financial and regulatory interventions to optimise use of CS [30]. Therefore, the primary data sources of this QCA are the intervention studies included in the Chen et al. [19] and Opiyo et al. [30]. To guide the analysis of the study, we used two qualitative evidence syntheses by Kingdon et al. [18, 28].

The intervention studies included in Chen et al. [19] and Opiyo et al. [30] are referred to as “cases” in this QCA. The main criteria to select eligible cases are the intervention should target healthcare providers and aim to reduce or optimise CS. We did not impose restrictions on study designs, therefore studies that were excluded in Chen et al. [19] and Opiyo et al. [30] due to study design (i.e. uncontrolled before and after study, interrupted time series with less than three data points) were re-assessed for eligibility in this analysis as it may help to show other pathways influencing success. We also assessed intervention studies published since the last review updates in 2018 and 2020, to ensure the inclusion of intervention studies that are likely included in future review updates.

To ensure that we have the most detailed and comprehensive information on each eligible case (intervention), we searched for sibling studies of eligible cases. Sibling studies (i.e. formative research, process evaluation) are studies that are linked to the main intervention study yet may have been published separately. Sibling studies can provide additional information about intervention components, study contexts, and implementation outcomes, which may not be sufficiently described in a single intervention effectiveness article. To locate the sibling studies, we conducted reference list search of Chen et al. [19], Opiyo et al. [30], all eligible cases, and Kingdon qualitative evidence syntheses [18, 28]. Additionally, we forward reference searched all the eligible cases using “Cited by” function in Scopus and Web of Science. Sibling studies were eligible for inclusion when they included any information on intervention components or implementation outcomes, regardless of methodology used. One review author (RIZ) screened all the potential sibling studies, and 10% of the screening was double checked by the second review author (MAB). Disagreements were discussed and adjudicated by the third review author, if needed.

In total, we identified 32 intervention studies targeting only healthcare providers or systems and six intervention studies targeting both women and healthcare providers or systems. The types of interventions targeting providers were comprised of audit and feedback (15 studies) [22, 33–46], financial reforms (11 studies) [47–57], implementation of second opinion without audit and feedback (2 studies) [23, 58], training to improve providers’ knowledge and skills (1 study) [59], introduction of collaborative midwifery and obstetrician models of care (1 study) [60], national publication of CS rates (1 study) [61] and legislatively-imposed practice guidelines (1 study) [62]. Due to an imbalance of successful and unsuccessful interventions in financial reform group (two successful [54, 55], 9 unsuccessful [47–53, 56, 57]), and a limited number of studies using interventions other than audit and feedback and financial reform (6/32 studies) [23, 58–62], these interventions could not be analysed (as QCA requires similar number of successful and unsuccessful interventions, typically at least 10 cases to be compared). Therefore, this QCA is based only on 15 audit and feedback interventions [22, 33–46] and six multi-target interventions [63–68].

#### Defining outcomes

Our primary outcome is “overall CS rate” in all women admitted for labour. Due to variation in outcome reporting, we categorized successful intervention (coded as 1) when the CS rate decreased and when a 95% confidence interval that did not cross the line of no effect or *p*-value ≤ 0.05; an unsuccessful intervention (coded was 0) was categorized when we observed that CS rate was increased or did not change.

#### Assessing risk of bias in main intervention studies

Risk of bias of included studies was considered throughout the study conduct. Risk of bias were reported for studies included in either Chen et al. [19] or Opiyo et al. [30] reviews, therefore we used their risk of bias results in this analysis. For studies that were not assessed by either Chen et al. [19] or Opiyo et al. [30], we assessed risk of bias using the same tools as used in the original review depending on their study design (Additional File 2 risk of bias assessment). We excluded studies assessed as high risk of bias, which may resulted us in missing information from relevant studies, yet necessary to ensure that this analysis is based on high quality studies and to allow researchers to develop deep case knowledge by limiting the overall number of included studies [69, 70].

### QCA stage 1: Identifying conditions, building data tables and calibration

 We identified potential conditions from the eligible cases using a combined deductive and inductive process. Firstly, we derived potential conditions deductively using our logic model (Additional File 1). Secondly, additional potential conditions were inductively derived from each eligible case using qualitative evidence “views” synthesis using Melendez-Torres’s approach [69] and intervention component analysis [71], where we examine potential conditions based on trialist’ reflections. The trialist’ reflections typically can be found in the discussion section of the paper and included contextual conditions like healthcare providers’ beliefs on CS, providers’ willingness to change, institutional culture, baseline CS rates, and policy relating CS. After consolidating similar conditions together, a total of 58 potential conditions were selected and extracted from each eligible case. Due to large numbers of potential conditions, we organized these conditions using a coding framework adapted from Harris et al. [25] to six main domains: 1) context and participants, 2) intervention design, 3) program content, 4) method of engagement, 5) health system factors, and 6) process outcomes (Additional File 3).

As the next step, we created the data table, which is a matrix where each eligible case is presented in a row and each potential condition in a column (Additional File 4). One author (RIZ) extracted conditions from each eligible case to the data table, which was then double reviewed by a second author (MVC or MAB). After the completion of the extraction, the extracted data needs to be calibrated before further analysis. The calibration (or often referred as coding) rules either using csQCA or fsQCA were developed based on the data and through consultations with all authors (Additional File 3). The calibration was then conducted using either direct or transformational assignment of qualitative and quantitative data [25, 27], to explore the extent to which interventions have ‘set membership’ with the outcome or conditions of interest. The calibration process was iterative, and the rules were revisited and re-defined based on the cases and literature.

### QCA stage 2: Constructing truth tables

 Once all data were calibrated, truth tables were constructed. Truth tables are an analytical tool in QCA to analyse associations between configurations of conditions and outcomes. Whereas the data table represents individual cases (rows) and individual conditions (columns) – the truth table synthesises this data to examine configurations – with each row representing a different configuration of the conditions. The truth tables indicate a) which conditions are featured in the configuration in that row; b) how many of the cases are represented by that configuration; and c) their association with the outcome.

Adhering to the “views synthesis as theory” perspective [69]*,* existing qualitative evidence syntheses and theoretical literature were used to guide the construction of truth tables. Our truth tables examined potential configurations of sufficient and necessary intervention, implementation and contextual conditions associated with a reduction in CS rates. After several iterations based on hypothesised theories about how the interventions should be delivered and assessment of the quality of the truth table, four final truth tables were constructed: 1) implementing training and education; 2) audit and feedback process; 3) multi-disciplinary collaboration; 4) consolidated model examining interactions of important conditions identified from models 1 to 3. Sub-analysis was also conducted to explore if similar conditions were observed in successful interventions in *interventions targeting both women and healthcare providers or systems* (“multi-target interventions”), among the components for providers only.

### QCA stage 3: Checking quality of truth tables

 As suggested by Thomas et al. [26], truth tables were iteratively developed, refined and improved through several measures. This includes assessing the number of studies contributing on each configuration, investigating the presence of contradictory results, and resolving any contradictions by considering theoretical perspectives. As there was an imbalance in the number successful and unsuccessful interventions, where the number of successful interventions was higher, we also conducted sensitivity analysis to see if the observed solutions found on our main solutions are similar or not when the number of successful and unsuccessful interventions were more balanced. In conducting the sensitivity analysis, we further selected studies which have provided impact of CS rate reduction among women in Robson group 1–4, as well as CS rate reduction in all women.

### QCA stage 4: Identifying parsimonious configurations through Boolean minimization

 The final truth tables were then simplified using Boolean minimization to explore simplified pathways observed in successful interventions. The initial solutions were “complex solutions”, which were then further minimized to the most “parsimonious solution” using R [27] which incorporates information about logical remainders (configurations where no cases were observed). We then explored intermediate solutions in which assumptions about logical remainders (e.g. those that are logically implausible) are specified by the analysts rather than by R [72].

### QCA stage 5: Checking the quality of the solution

 We checked the quality of the solutions by checking consistency (i.e. the proportion of cases with a particular configuration that are associated with the outcome of interest) and coverage scores (i.e. the proportion of cases in the outcome set that are supported or ‘covered’ by cases with a particular configuration or condition) as well as by analysing configurations associated with the negation of the outcome to see if it predicts the observed solutions. As the final consolidated solution, we present the intermediate solution instead of the parsimonious solution, as it is the most logical and closely aligned with the real-world settings.

### QCA stage 6: Interpretation of solutions

 We interpreted the results iteratively through discussion among all authors. We adopted this reflexive approach to ensure that the interpretation is aligned with the theoretical and research literature, possible clinical pathways, methodological approaches, and coherent with current understandings of the phenomenon.

## Results

### Overview of included studies

**Fig. 1:**
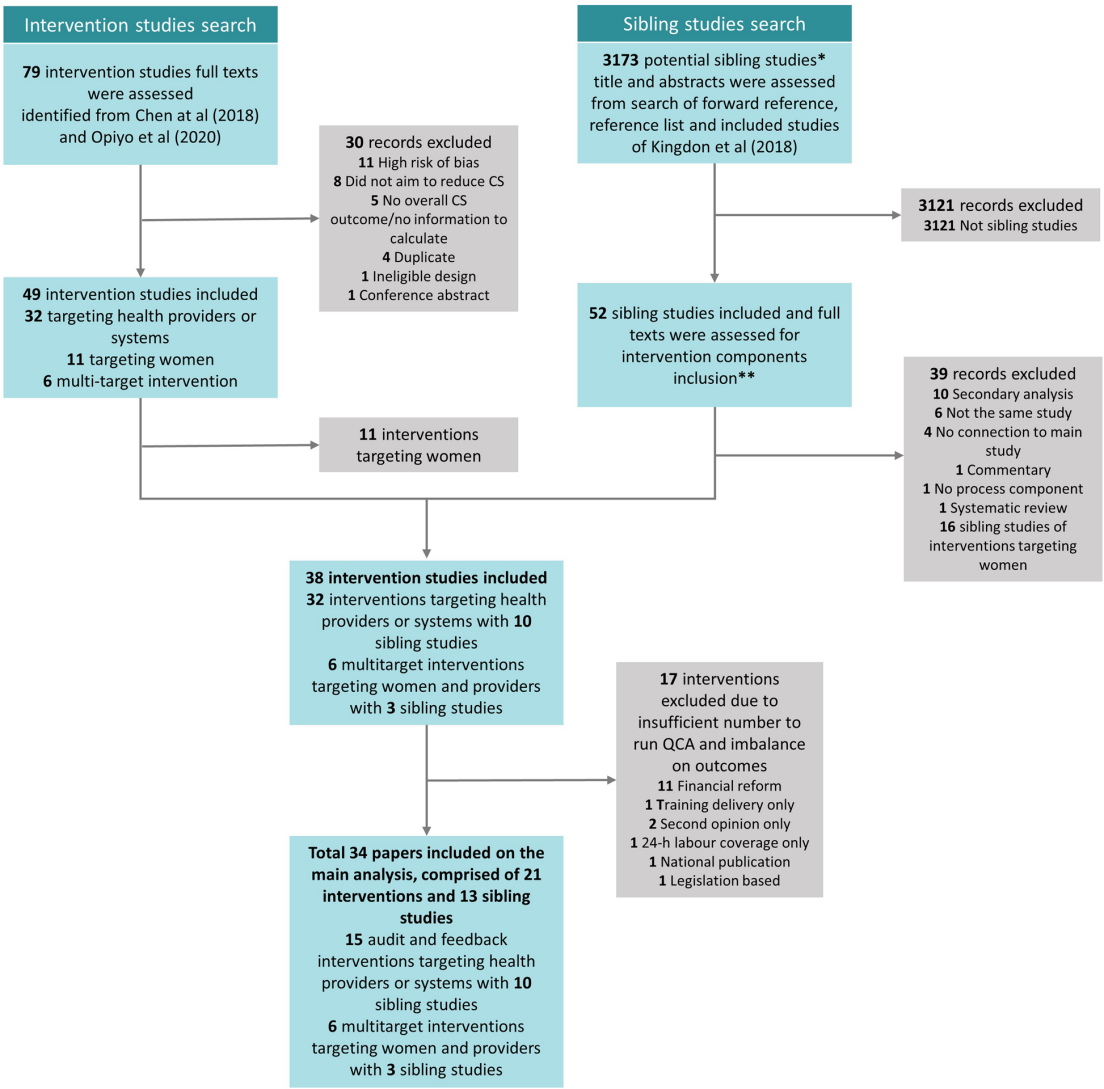
PRISMA flowchart. This figure depicts the PRISMA flow diagram, detailing the intervention and sibling studies searches, number of abstracts and full texts screened, reasons for exclusion, and included studies and papers. *Sibling studies: studies which were conducted on the same settings, participants, and timeframe. **Intervention components: information on intervention input, activities, outputs, including intervention context and other characteristics

This QCA is based on 15 audit and feedback interventions targeting healthcare providers [22, 33–46] and six interventions targeting both healthcare providers and women (multi-target interventions) [63–68]. The interventions are reported in 34 papers, comprising of 21 intervention evaluation studies and 13 sibling studies (See Fig. 1: PRISMA Flowchart). Table 1 (summarised version) and Additional File 5 (full version) show characteristics of included studies.

**Table 1:** Summary of selected characteristics of studies included with interventions focused on audit and feedback

Study characteristics	n (%) *n* = 21	References
**Setting**		
**High income countries**	12 (57.1%)	[22, 24, 33, 38–44, 67, 73]
**Middle-income countries**	8 (38.1%)	[34, 45, 46, 63–66, 68]
**Low-income countries**	1(4.8%)	[35]
**Data collection period**		
**< 2000**	7 (33.3%)	[22, 33, 38, 39, 43, 44, 73]
**2000–2010**	5 (23.8)	[40–42, 45, 46]
**> 2010**^a^	9 (42.9%)	[24, 34, 35, 63–68]
**Study design**		
**Randomized controlled trial**	5 (23.8%)	[24, 33, 35, 67, 68]
**Before and after**	7 (33.3%)	[40, 42, 45, 46, 63, 64, 66]
**Interrupted time series**	8 (38.1%)	[22, 34, 38, 39, 41, 43, 44, 65]
**Retrospective cohort**	1 (4.8%)	[73]
**Type of women**		
**All pregnant women**	14 (66.7%)	[22, 24, 33–35, 38–40, 42–46, 73]
**Women with low-risk pregnancy**	6 (28.6%)	[41, 63–66, 68]
**Women with previous CS**	1 (4.8%)	[67]
**Baseline CS rate**		
**< 20%**	3 (14.3%)^b^	[22, 33, 42]
**20–39%**	7 (33.3%)^b^	[24, 35, 39, 40, 42, 44, 73]
**30–39%**	3 (14.3%)	[38, 41, 43]
**≥ 40%**	9 (42.9%)	[34, 45, 46, 63–68]
**Outcomes**		
**Successful interventions**	14 (66.7%)	[22, 24, 33–35, 38–40, 63–67, 73]
**Unsuccessful interventions**	7 (33.3%)	[41–46, 68]

^a^Study started before 2010 and ended after 2010 is categorised as > 2010

^b^One study [42] involved facilities which have two different CS rates

The 15 studies of interventions targeting healthcare providers comprised of nine successful [22, 33–40] and six unsuccessful interventions [41–46] in reducing CS. Ten sibling studies were identified [37, 74–82], associated with six intervention studies [24, 33–35, 45, 73]. The 15 intervention studies were conducted in seven regions: North America (5 from United States of America [22, 38, 39, 44, 73], 2 from Canada [24, 33]), Latin America and Caribbean (1 from Chile [41]), Asia and Pacific (1 From Malaysia [45], 1 from Taiwan [43], 1 from India [34]), Europe (1 from Spain [42], 1 from Netherlands [40]), Middle East & North Africa (1 from Iran [46]), Sub-Saharan Africa (1 from Burkina Faso [35]). These countries comprised of 11 high-income, three middle-income countries, and one low-income country. Six studies were included in Chen et al. in which one was graded as low risk of bias [24] and five graded as having some concerns [33, 41, 43, 44, 46], two studies were newly published studies graded as having some concerns [34, 35], and the remaining seven were excluded studies of Chen et al. and graded as having some concerns [22, 38–40, 42, 45, 73].

The six multi-target intervention studies consisted of five successful interventions [63–67] and one unsuccessful intervention [68]. Three sibling studies [83–85] were identified from one multi-target intervention [67]. The interventions were delivered across three regions: Latin America and Caribbean (1 from Brazil [65]), Asia and the Pacific (4 from China [63, 64, 66, 68]), Europe (1 from Italy [67], 1 from Ireland [67], and 1 from Germany [67]). One study was conducted in a high-income country, while five studies were conducted in an upper middle-income country (four from China). Out of the six intervention studies, one intervention study was included in Chen et al. [19] was graded as having some concerns [66], two studies included in Opiyo et al. [30] were graded as having not serious concerns [64, 65], and three studies were newly published where one study assessed as having not serious concerns [63] and two studies assessed as having some concerns on their risk of bias [67, 68] (Additional File 2).

Among the audit and feedback interventions targeting healthcare providers, only four used theory or programme theory to inform intervention design [22, 41, 45, 73], while the remaining referred to previous intervention studies as the basis for intervention design, or did not refer to other evidence to inform intervention design. The four theory-based interventions adopted either Robson ten groups classification system [22, 41], quality improvement framework [73], plan-act-reflect cycle [45], or the logical framework [45] to guide intervention design. Audit and feedback was often accompanied by other interventions, including implementation of guidelines or protocols for CS indications [24, 34, 35, 39–44, 46, 82], training to improve providers’ knowledge and skills [24, 34, 35, 38, 42, 43, 73], mandatory second opinion [38, 43], opinion leaders [24, 33, 43, 45], or implementation of collaborative midwifery and obstetrician models of care [44]. Four interventions used top-down enforcement or were “dictated” in nature [42, 43, 46, 73], while the remaining were “reflective”, meaning that interventions leveraged bottom-up approach through discussions and consultations. Training for healthcare providers typically focused on improving knowledge and skills on antenatal and labour management (i.e. fetal monitoring training, perineal massage, external cephalic version), CS indications, and the purpose of intervention. Eight intervention studies promoted multi-disciplinary collaboration between obstetricians, midwives, nurses, and other doctors [22, 24, 34, 35, 40, 41, 73]. The six multi-target audit and feedback interventions typically also included training for healthcare providers [63–68] and dissemination of CS indications [64, 66], while the intervention component targeting women consisted of childbirth education [63–68]. Across all 21 studies, there were very limited data on implementation outcomes such as adherence, attrition, fidelity, and satisfaction.

### Qualitative comparative analysis of the audit and feedback interventions

We conducted six analyses (‘models’) to explore factors leading to successful interventions”. The first three (models 1–3) assessed different aspects of audit and feedback interventions within the 15 interventions targeting healthcare providers only. These models were developed based on theoretical rationales from views synthesis (Table 2). The fourth ‘consolidated model’ brought together important conditions from model 1–3. The fifth model also included the six multi-target cases to explore if conditions from the consolidated model were also observed in the interventions targeting both women and healthcare providers. Finally, the sixth model was a sensitivity analysis to confirm that the imbalance in number between successful and unsuccessful cases among the 15 interventions targeting healthcare providers only did not skew results. The definitions of conditions used in constructing truth tables are depicted in Table 3. Some of the conditions may overlap between the models, as views synthesis indicated that certain component could be important on intervention targeting health providers (e.g. willingness to change, dictated nature of intervention). It is important to note that the referred “important” conditions on this paper are *sufficient*, not necessary, conditions in prompting successful interventions.

**Table 2:** Views synthesis driving the construction of model 1–3 and its truth tables

**Model 1 – Implementing training to improve providers knowledge and clinical skills**
The existing qualitative evidence synthesis and intervention component analysis indicated four different themes relating to training [18, 28, 34, 44, 86]. Firstly, healthcare providers are often reluctant to implement new CS programs or to implement overall change due to perceived insufficiency of skills and knowledge on labour and vaginal birth management, especially the younger generation [28]. Secondly, healthcare providers and other stakeholders (i.e. policy makers, hospital managers) emphasised the importance of implementing various training or education for healthcare providers [18, 28, 86]. This training includes clinical skills training in labour and vaginal birth, recommendations in practice, clinical audit and program content itself [18, 28, 86]. Thirdly, both providers [28, 86] and trialists [34, 44] mentioned that the underlying factor of success lies on providers’ beliefs about CS and vaginal birth, as well as whether providers are willing to step out of their comfort zone to. Lastly, providers mentioned that they preferred the intervention to be reflective in nature, instead of dictatorial and enforcing [28]
**Model 2 – The audit and feedback process**
In relation to audit and feedback process, the existing qualitative evidence synthesis and intervention component analysis revealed three different themes [28, 86]. Firstly, the process of conducting audit and feedback was considered critical by healthcare providers, as the content, methods of delivery, and timing of audit and feedback influenced how they feel about the intervention overall [86]. Secondly, some providers were concerned that audit and feedback may pose a threat to their identities and careers [28]. Therefore, the more acceptable the structure of feedback is to the providers (i.e. feedback delivered individually instead in group), the better they respond to it, thus increasing its effectiveness [86]. Thirdly, findings by Kingdon et al. also revealed organisations which were able to reduce CS are often characterised by having healthcare providers who valued continuous quality improvements, such as clinical audits, second opinion, continuing education [28]
**Model 3 – Working relationship and environment**
In terms of working relationship and environment, existing qualitative evidence syntheses by Kingdon et al. revealed three themes [18, 28]. Firstly, multi-disciplinary collaboration between doctors, midwives, nurses and other maternity care providers was pointed as a key element in optimising CS [18, 28]. Multi-disciplinary collaboration has been observed as very poor in health facilities with high CS rates, and actively present in health facilities with low CS rates [18, 28]. Secondly, healthcare providers reported about the unequal and hierarchical power relations when caring for women. Working relationships, collaboration and communication may also be diminished through hierarchy-driven fear, which may be present when for example midwives are considered to have fewer skills than doctors [18, 28]. Thirdly, Kingdon et al. also emphasized the effectiveness of interventions to reduce unnecessary CS is strongly mediated by stakeholder commitment and organizational buy-in or, systems and policy changes that facilitate vaginal birth [18]

**Table 3:** Operational definitions of conditions used in constructing truth tables

**Actionable recommendations:** each audit and feedback cycle produced actionable recommendations that healthcare providers could act upon until next cycle
**Active dissemination of CS indications**: implementation CS indications, such as clinical algorithms on when to conduct CS, through guidelines or protocol implementation, information, education and communication (IEC) materials, or reminder systems)
**Dictated nature of intervention:** intervention which used top-down enforcement where mandate to reduce CS was imposed
**Frequent audit and feedback cycle:** frequent audit and feedback cycle which classified either weekly or monthly
**Healthcare providers’ willingness to change:** providers willingness to adopt to change and adhere to the intervention. Willingness to change was added as it becomes and overarching factor across qualitative evidence syntheses [28, 86] and discussion section of trials reports [34, 44] – where both providers and trialists mentioned that the underlying factor of success lies on providers’ beliefs about CS and vaginal birth, as well as whether providers are willing to step out of their comfort zone to change
**Individual dissemination of audit and feedback results:** dissemination of audit and feedback results to providers individually instead in group settings
**Internal policies that support vaginal birth:** whether internal policies that support vaginal birth or the intervention exists outside of the intervention. This include national consensus in improving CS rates where CS is nationally treated as a measure of institutional and individual practice quality [44], recommended maternity practices supporting physiologic birth [39, 41, 43, 45], national guidelines on vaginal birth after caesarean (VBAC) [67], equipment and technical support for local healthcare facilities [63], implementation of new care models favouring physiologic birth [65], additional rooms to support physiologic birth [65], hire full-time obstetricians [34], and increase staffing in the labour ward [34]
**Multidisciplinary collaboration:** when the intervention involved different cadre of health workers in caring for women, which could include team of obstetricians, midwives, nurses, and doctors working together
**Reflective nature of intervention:** Leveraged bottom-up approach through discussions and consultations
**Training to improve providers’ knowledge and skills**: implementation of theory-based or practical education session for healthcare providers to improve their knowledge and skills on labour management

#### Model 1 – Implementing training to improve providers knowledge and clinical skills (*n* = 15 cases)

Based on the views syntheses as seen on Table 2, we constructed a truth table using csQCA with 15 interventions targeting healthcare providers only by considering four different conditions relating to training: 1) training to improve providers’ knowledge and skills, 2) active dissemination of CS indications, 3) healthcare providers’ willingness to change, 4) dictated nature of the intervention.

Out of 16 possible configurations, we identified eight configurations (Table 4). The first four rows depict the configurations of successful interventions with perfect consistency (inclusion score = 1), while the remaining four rows are configurations of unsuccessful interventions. Among the configurations of successful interventions, when both training and active dissemination of CS indications are implemented (row 1), or either training or active dissemination of CS indications are implemented in a context where providers’ show willingness to change (row 2 and 3), dictated nature is not needed to prompt successful outcomes. However, when only training is present without other intervention or contextual conditions, dictated nature is necessary to achieve successful outcomes (row 4). Unsuccessful interventions were characterised by consistent absence of willingness to change by providers (row 5–8), adopted dictated nature of intervention in the presence of training and active dissemination of CS indications (row 5), and when only active dissemination of CS indications (without training) present (row 7).

**Table 4:** Truth table model 1. The table demonstrates the truth table results from model 1—implementing training to improve providers knowledge and clinical skills based on 15 intervention studies. The truth table shows the associations between configurations of conditions and outcomes

Row	Training to improve knowledge and skills	Active dissemination of CS indications	Providers’ willingness to change	Dictated	CS outcome	Number of studies	Inclusion score^a﻿^	PRI^b^	Cases
1	1	1	0	0	1	2	1	1	[24, 35]
2	0	1	1	0	1	3	1	1	[33, 39, 40]
3	1	0	1	0	1	3	1	1	[22, 34, 38]
4	1	0	0	1	1	1	1	1	[73]
5	1	1	0	1	0	2	0	0	[42, 43]
6	0	1	0	0	0	2	0	0	[41, 44]
7	0	1	0	1	0	1	0	0	[46]
8	1	0	0	0	0	1	0	0	[45]

^a^Inclusion score: sometimes referred as consistency indicates the degree to which the evidence is consistent with the hypothesis that there is sufficiency relation between the configuration and the outcome; ^b^PRI: Proportional Reduction in Inconsistency, refers to the extent in which a configuration is sufficient in triggering successful outcome as well as the negation of the outcome

**Fig. 2:**
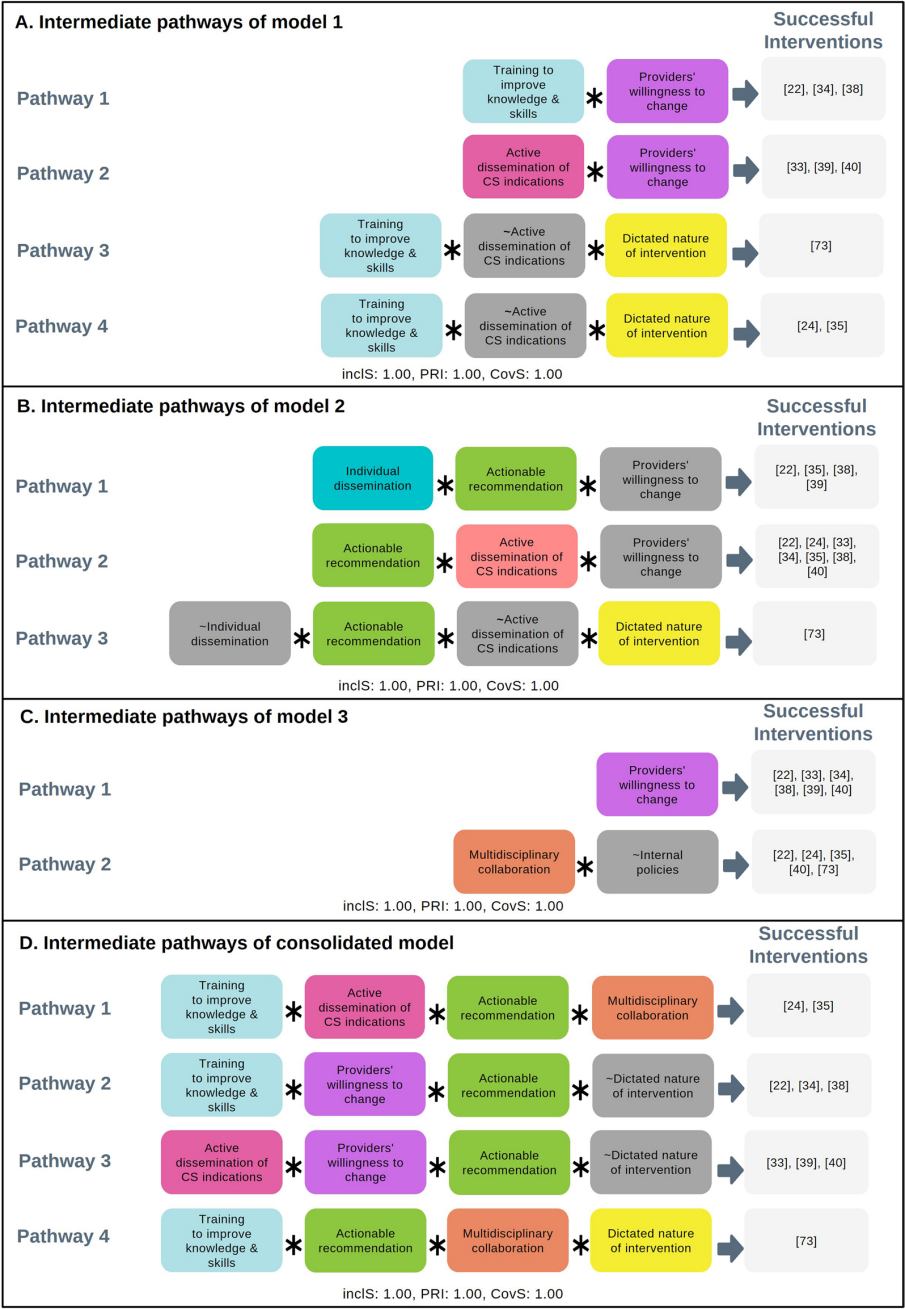
Intermediate pathways or solutions that trigger successful audit and feedback interventions to optimise CS. This panel depicts four figures showing intermediate pathways that lead to successful reduction of CS from four models of analysis, that is model 1–3 and consolidated model. Grey box with “ ~ ” notation referred to absence of such condition; Inclusion score (InclS) sometimes referred as consistency indicates the degree to which the evidence is consistent with the hypothesis that there is sufficiency relation between the configuration and the outcome; PRI stands for Proportional Reduction in Inconsistency and refers to the extent in which a configuration is sufficient in triggering successful outcome as well as the negation of the outcome; Coverage score (CovS) refers to percentage of cases for which the configuration is valid

Boolean minimisation revealed four pathways to successful interventions (Fig. 2A). The first two pathways show that when there is providers’ willingness to change and either training to improve providers’ knowledge and skills or active dissemination of CS indications, successful outcomes are observed. However, when there is no providers’ willingness to change and active dissemination of CS indications, it is important to implement training and dictated nature of intervention at the same time to trigger successful intervention (third pathway). Lastly, the fourth pathway shows that dictated nature of intervention is not needed when both training and active dissemination of CS indications are present at the same time to prompt successful intervention. These solutions show that all the four conditions seem to play a role in influencing intervention success: providers’ willingness to change, dictated nature of intervention, training to improve knowledge and skills, and active dissemination of CS indications.

#### Model 2 – The audit and feedback process (*n* = 15 cases)

When constructing this truth table using csQCA, four conditions relevant to the structure of audit and feedback were included: 1) frequent audit and feedback cycle, 2) individual dissemination of audit and feedback results, 3) actionable recommendations, and 4) dictated nature of intervention.

Out of 16 possible configurations, we observed nine configurations (Table 5). The first four rows show the configurations of successful conditions with perfect consistency (inclusion = 1), while the remaining five rows show configurations of unsuccessful interventions. From the truth tables, among the successful interventions, the presence of actionable recommendations with dictated nature of intervention in the absence of frequent audit and feedback cycle and individual dissemination (row 1), or the presence of actionable recommendations and frequent audit and feedback cycle in the absence of dictated and individual dissemination (row 2), prompt successful implementation. Successful implementation was also prompted when actionable recommendations and individual dissemination were present at the same time either in the absence (row 4) or presence (row 3) of frequent audit and feedback cycle. Unsuccessful interventions were characterised by the absence of all the conditions above (row 5), the absence of actionable recommendations (row 7–9), as well as the present of dictated nature of intervention when actionable recommendations and frequent audit and feedback cycle are present (row 6).

**Table 5:** Truth table model 2. The table demonstrates the truth table results from model 2 – audit and feedback process based on 15 intervention studies. The truth table shows the associations between configurations of conditions and outcomes

Row	Individual dissemination	Actionable recommendations	Frequency of audit and feedback cycle	Dictated	CS outcome	Number of studies	Inclusion score^a^﻿	PRI^b^	Cases
1	0	1	0	1	1	1	1	1	[73]
2	0	1	1	0	1	4	1	1	[24, 33, 34, 40]
3	1	1	0	0	1	1	1	1	[39]
4	1	1	1	0	1	3	1	1	[22, 35, 38]
5	0	0	0	0	0	1	0	0	[45]
6	0	1	1	1	0	2	0	0	[42, 43]
7	1	0	0	0	0	1	0	0	[44]
8	1	0	1	0	0	1	0	0	[41]
9	1	0	1	1	0	1	0	0	[46]

^a^Inclusion score: sometimes referred as consistency indicates the degree to which the evidence is consistent with the hypothesis that there is sufficiency relation between the configuration and the outcome; ^b^PRI: Proportional Reduction in Inconsistency, refers to the extent in which a configuration is sufficient in triggering successful outcome as well as the negation of the outcome

Boolean minimisation identified three intermediate pathways (Fig. 2B). The first and second pathways show that despite the absence of dictated nature of the intervention, the presence of actionable recommendations and individual dissemination, or actionable recommendations with frequent audit and feedback cycle, prompt successful implementation. The last pathway shows that when individual dissemination and frequent audit and feedback cycle are absent, successful implementation can only be triggered when actionable recommendations and dictated nature of intervention are present. From these solutions, we conclude that actionable recommendations work jointly with other conditions (frequent audit and feedback cycle, individual dissemination or dictated nature of intervention) in influencing success.

#### Model 3 – Working relationship and environment (*n* = 15 cases)

Utilising csQCA, this truth table was constructed by including three conditions in relation to working relationships and environment: 1) multidisciplinary collaboration, [2] healthcare providers’ willingness to change, and 3) internal policies that support vaginal birth.

Out of eight possible configurations, we identified eight configurations, comprising of five configurations with successful interventions with perfect consistency (inclusion score = 1), and three configurations with unsuccessful interventions (Table 6). The presence of all conditions prompt successful intervention as shown on row 1. On the second and third row, we can see that successful outcomes are observed where willingness to change is present in combination with another condition, including presence of multidisciplinary collaboration (row 2) and internal policies (row 3). Interestingly, the presence of only multi-disciplinary collaboration or only providers’ willingness to change in the absence of other conditions also triggers successful intervention (row 4 & 5), noting that the cases are comparatively older studies (1996, 1998) [22, 38] when social, medical and legal pressure may have not been (or may have not been perceived) as strong as at present. Unsuccessful interventions were consistently characterised by the absence of all the conditions above (row 7) and willingness to change (row 6–7).

**Table 6:** Truth table model 3. The table demonstrates the truth table results from model 3 – working relationships and environment based on 15 intervention studies. The truth table shows the associations between configurations of conditions and outcomes

Row	Multidisciplinary collaboration	Providers' willingness to change	Internal policies	CS outcome	Number of studies	Inclusion score^a^	PRI^b^	Cases
1	1	1	1	1	1	1	1	[34]
2	1	1	0	1	2	1	1	[22, 40]
3	0	1	1	1	1	1	1	[39]
4	1	0	0	1	3	1	1	[24, 35, 73]
5	0	1	0	1	2	1	1	[33, 38]
6	0	0	0	0	2	0	0	[42, 46]
7	0	0	1	0	2	0	0	[43, 44]
8	1	0	1	0	2	0	0	[41, 45]

^a^Inclusion score: sometimes referred as consistency indicates the degree to which the evidence is consistent with the hypothesis that there is sufficiency relation between the configuration and the outcome; ^b^PRI: Proportional Reduction in Inconsistency, refers to the extent in which a configuration is sufficient in triggering successful outcome as well as the negation of the outcome

Boolean minimisation identified two intermediate pathways (Fig. 2C). On the first pathway, providers’ willingness to change results in successful interventions. On the second pathway, when internal policies supporting vaginal birth is missing, the presence of multidisciplinary team collaboration results in successful intervention. From this solutions, providers’ willingness to change and multidisciplinary collaboration seem to be important in influencing success.

#### Consolidated model – Important conditions to prompt successful interventions targeting healthcare providers (*n* = 15 cases)

We consolidated the learning from the three models explored above to find the final important conditions that prompt successful interventions targeting healthcare providers. In constructing this consolidated model, we included the important conditions identified in the first three models explored. These important conditions are 1) training to improve providers’ knowledge and skills, 2) active dissemination of CS indications, 3) providers’ willingness to change, 4) actionable recommendations, 5) multidisciplinary collaboration, and 6) dictated nature of intervention.

Out of 64 possible configurations, 11 configurations are observed, consisting of 6 successful configurations with perfect consistency (inclusion score = 1) and 5 unsuccessful configurations (Table 7). Boolean minimisation revealed four pathways to success (Fig. 2D). The first pathway shows that the presence of training to improve providers’ knowledge and skills, active dissemination of CS indications, actionable recommendations, and multidisciplinary collaboration prompt successful intervention. On the second and third pathway, we can see that the presence of training or active dissemination of CS indications combined with providers’ willingness to change and actionable recommendations prompt successful intervention, even in the absence of dictated nature of intervention. Interestingly, in the last pathway, when providers’ willingness of change and active dissemination of CS indications seems to be absent, dictated nature intervention is necessary alongside the implementation of training, actionable recommendations, and multidisciplinary collaboration to trigger successful intervention.

**Table 7:** Truth table of consolidated model. The table demonstrates the truth table results from model 4 – consolidated model based on 15 intervention studies. The truth table shows the associations between configurations of conditions and outcomes

Row	Training to improve knowledge and skills	Active dissemination of CS indications	Providers’ willingness to change	Actionable recommendations	Multidisciplinary collaboration	Dictated	CS outcome	Number of studies	Inclusion score^a^	PRI^b^	Cases
1	0	1	1	1	0	0	1	2	1	1	[33, 39]
2	0	1	1	1	1	0	1	1	1	1	[40]
3	1	0	1	1	1	0	1	2	1	1	[22, 34]
4	1	0	0	1	1	1	1	1	1	1	[73]
5	1	0	1	1	0	0	1	1	1	1	[38]
6	1	1	0	1	1	0	1	2	1	1	[24, 35]
7	0	1	0	0	0	0	0	1	0	0	[44]
8	0	1	0	0	0	1	0	1	0	0	[46]
9	0	1	0	0	1	0	0	1	0	0	[41]
10	1	0	0	0	1	0	0	1	0	0	[45]
11	1	1	0	1	0	1	0	2	0	0	[42, 43]

^a^Inclusion score: sometimes referred as consistency indicates the degree to which the evidence is consistent with the hypothesis that there is sufficiency relation between the configuration and the outcome; ^b^PRI: Proportional Reduction in Inconsistency, refers to the extent in which a configuration is sufficient in triggering successful outcome as well as the negation of the outcome

From these solutions, we identified five important components that may prompt successful intervention 1) provide training to improve providers’ knowledge and skills, 2) active dissemination of CS indications, 3) actionable recommendations, 4) leverage multidisciplinary collaboration, and 5) providers’ willingness to change. Importantly, when one or more of these components are absent (especially willingness to change and training or active dissemination of CS indications), dictated nature of intervention is needed to prompt successful interventions.

### Sub-analysis – Interventions targeting both women and healthcare providers or systems (*n* = 21 cases)

We conducted a sub-analysis to explore if similar important conditions are observed in the interventions targeting both women and healthcare providers. In doing this analysis, we have included an additional six intervention studies targeting both women and healthcare providers, therefore 21 studies were included in this analysis. For this model, the six conditions identified from the consolidated model plus and additional ‘multi-target intervention’ condition were used to run the truth tables using csQCA (Table 8).

**Table 8:** Truth table of sub-analysis with multi-target interventions. The table demonstrates the truth table results from model 5 – sub-analysis with multi-target interventions based on 21 intervention studies. The truth table shows the associations between configurations of conditions and outcomes

Row	Training to improve knowledge and skills	Active dissemination of CS indications	Providers' willingness to change	Actionable recommendations	Multidisciplinary collaboration	Dictated	Multi-target interventions	CS outcome	Number of studies	Inclusion score^a^	PRI^b^	Cases
1	0	1	1	1	0	0	0	1	2	1	1	[33, 39]
2	0	1	1	1	1	0	0	1	1	1	1	[40]
3	1	0	0	1	1	1	0	1	1	1	1	[73]
4	1	0	0	1	1	1	1	1	3	1	1	[63, 65, 67]
5	1	0	1	1	0	0	0	1	1	1	1	[38]
6	1	0	1	1	1	0	0	1	2	1	1	[22, 34]
7	1	1	0	1	0	1	1	1	2	1	1	[64, 66]
8	1	1	0	1	1	0	0	1	2	1	1	[24, 35]
9	0	1	0	0	0	0	0	0	1	0	0	[44]
10	0	1	0	0	0	1	0	0	1	0	0	[46]
11	0	1	0	0	1	0	0	0	1	0	0	[41]
12	1	0	0	0	1	0	0	0	1	0	0	[45]
13	1	0	0	1	0	1	1	0	1	0	0	[68]
14	1	1	0	1	0	1	0	0	2	0	0	[42, 43]

^a^Inclusion score: sometimes referred as consistency indicates the degree to which the evidence is consistent with the hypothesis that there is sufficiency relation between the configuration and the outcome; ^b^PRI: Proportional Reduction in Inconsistency, refers to the extent in which a configuration is sufficient in triggering successful outcome as well as the negation of the outcome

**Fig. 3:**
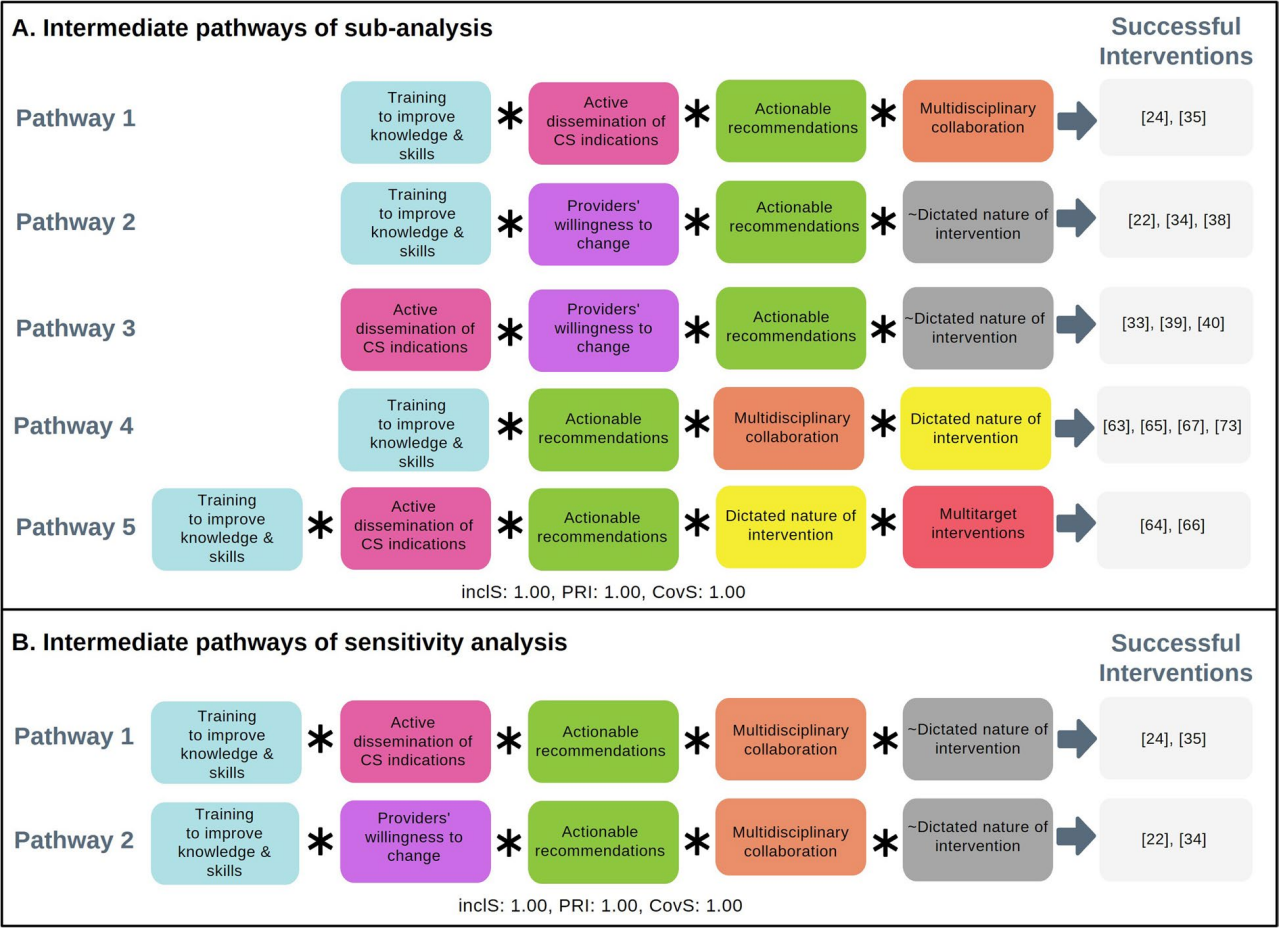
Intermediate pathways or solutions from sub-analysis and sensitivity analysis. This panel depicts two figures showing intermediate pathways that lead to successful reduction of CS from two models of analysis, that is sub-analysis and sensitivity analysis models. Grey box with “ ~ ” notation referred to absence of such condition; Inclusion score (InclS) sometimes referred as consistency indicates the degree to which the evidence is consistent with the hypothesis that there is sufficiency relation between the configuration and the outcome; PRI stands for Proportional Reduction in Inconsistency and refers to the extent in which a configuration is sufficient in triggering successful outcome as well as the negation of the outcome; Coverage score (CovS) refers to percentage of cases for which the configuration is valid

Boolean minimisation reveals similar intermediate pathways with the consolidated model of interventions targeting healthcare providers only (Fig. 3A). The only difference is that among multi-target interventions only (pathway 5), in the absence of providers’ willingness to change and multidisciplinary collaboration, dictated nature of intervention is needed alongside training to improve knowledge and skills, active dissemination of CS indications, and actionable recommendations to prompt successful interventions. However, more investigation is needed to examine interactions between components targeting women and providers.

### Sensitivity analysis (*n* = 15)

We conducted sensitivity analysis as there is a modest imbalance between successful (*n* = 9) and unsuccessful interventions (*n* = 6) in the data for the main analysis. The sensitivity analysis was conducted to explore if the observed solutions in the main consolidated solutions are similar or not when the number of successful and unsuccessful interventions are more balanced.

Out of nine successful interventions, we selected studies which successfully decreased CS among healthy women in Robson group 1–4 (e.g. women with term, singleton, cephalic pregnancies without previous CS. A total of four studies successfully decreased CS in Robson group 1–4 [22, 24, 34, 35]. Therefore, 10 studies were included for the sensitivity analysis, comprising of four successful interventions [22, 24, 34, 35] and six unsuccessful interventions [41–46], where the same six conditions from consolidated model were used (Table 9).

**Table 9:** Truth table of sensitivity analysis. The table demonstrates the truth table results from model 6 – sensitivity analysis based on 15 intervention studies. The truth table shows the associations between configurations of conditions and outcomes

Row	Training to improve knowledge and skills	Active dissemination of CS indications	Providers' willingness to change	Actionable recommendations	Multidisciplinary collaboration	Dictated	CS outcome	Number of studies	Inclusion score^a^	PRI^b^	Cases
1	1	0	1	1	1	0	1	2	1	1	[22, 34]
2	1	1	0	1	1	0	1	2	1	1	[24, 35]
3	0	1	0	0	0	0	0	1	0	0	[46]
4	0	1	0	0	0	0	0	1	0	0	[44]
5	0	1	0	0	1	0	0	1	0	0	[41]
6	1	0	0	0	1	0	0	1	0	0	[45]
7	1	1	0	1	0	1	0	2	0	0	[42, 43]

^a^Inclusion score: sometimes referred as consistency indicates the degree to which the evidence is consistent with the hypothesis that there is sufficiency relation between the configuration and the outcome; ^b^PRI: Proportional Reduction in Inconsistency, refers to the extent in which a configuration is sufficient in triggering successful outcome as well as the negation of the outcome

Overall, the sensitivity analysis supports the main analysis results (consolidated model), as the sensitivity analysis shows that when all conditions except dictated nature of intervention are present, it prompts successful interventions (Fig. 3B). However, when some components are absent, dictated nature of intervention is needed to prompt successful interventions, as shown on the main analysis results.

## Discussion

Our QCA aimed to explore important intervention components which can trigger the success in optimizing CS use under the umbrella of audit and feedback interventions. Through the consolidated model, the QCA revealed successful audit and feedback interventions targeting healthcare providers were characterised by the presence of training to improve providers’ knowledge and skills, active dissemination of CS indications, actionable recommendations, multidisciplinary collaboration, and providers’ willingness to change. Importantly, when one or more of these components are absent (especially willingness to change and training or active dissemination of CS indications), adoption of dictated nature in the intervention is needed to trigger successful interventions. These important conditions do not work in silos, but work jointly as parts of configurations to enable successful interventions.

Willingness to change was shown to be one of the sufficient conditions driving the success of intervention. Previous studies have shown that willingness to change influence participants’ adherence to the intervention [28, 86]. This is aligned with the theory of planned behaviour, which links individual beliefs, norms, and attitudes to intentions and behaviours [87]. In the context of audit and feedback, some providers reported doubts about the intervention and alignment to their priorities as cause on the reluctancy to engage and adhere to intervention [86]. Specifically for CS, providers were also sometimes unwilling to engage with change due to concerns about potential loss of income, threats to professional status if litigation occurs, and differing values and beliefs about CS provision [18, 28]. Irrespective of the root causes of provider unwillingness to change, when unwillingness to change is present, it is more difficult to encourage intervention adherence and sustainability.

Kingdon et al. stated that while individual willingness to change is important, it may not be sustainable, especially when factors influencing change from social, organisational and system levels are not addressed [18]. Interestingly from our QCA, willingness to change is closely tied to training and active dissemination of CS indications. When willingness to change is absent, our QCA shows that training and active dissemination of CS indications should be present to prompt successful intervention. Existing research on behaviour change interventions suggests that training and information dissemination improved knowledge and professional competency, which then improved decision-making and clinical outcomes [88]. However, research also shows that training and information alone is not sufficient in many cases to change behaviour [89, 90], which supports the configurations of sufficient conditions on our QCA. While exploring the critical elements of training and dissemination were of interest in this QCA (i.e. frequency, duration, mode of interaction, practice sessions), there was insufficient detail in the included studies to conduct this analysis.

We also found that continuity of action is an important condition to trigger successful intervention alongside the other components. From our study, we found two differing types of audit and feedback in relation to continuity: 1) audit and feedback were only done to evaluate providers’ adherence and 2) audit and feedback were also used to produce actionable recommendations and implement continuous improvement action [24, 34, 35]. We found the latter to be important in influencing success of the intervention. For example, in one study the first audit and feedback cycle produced new clinical protocol and guideline as an action, and the next cycles resulted in implementation of training programs followed by improvement of labour wards, strengthened antenatal class and improvement collaboration participation [34]. Continuity of action seems to be important in the context of CS intervention as it introduces changed of culture, specifically “culture of continuous improvement”, at organisation level which directly influence individuals by promoting learning [18, 91]. Furthermore, Foy et al. (2020) also proposes that the success of audit and feedback depends on the clear actionable messages for both the organisations and individuals, “emphasising action over measurement” [92]. Therefore, ensuring actionable recommendations in audit and feedback is crucial in ensuring benefits.

Hierarchies and imbalanced power relations are common in clinical settings and can create communication barriers as well as marginalisation of midwives, nurses and junior doctors from decision-making that may affect care decisions [18, 28]. To address this, multidisciplinary collaboration [18, 28, 93] was introduced in some studies, and our QCA results show that multidisciplinary collaboration prompts successful reductions of CS together with other components mentioned above. One of the reasons why multidisciplinary collaboration reduced CS is more related to the general atmosphere and ethos which were built when it was leveraged: strong teamwork where every member in the team has greater awareness on what their roles are, work collaboratively in resolving issues, and communicate with each other respectfully. For example, a recent study in Brazil reported that implementing multidisciplinary collaboration among providers, along with engagement with pregnant women and improvements to hospital facilities reduced CS and increased vaginal birth [20].

Whether the intervention should be dictatorial (top down) or reflective (bottom up) in nature is a delicate balance. While top-down dictatorial interventions can direct change, reflective, participatory, or bottom-up interventions shift the power to people to drive the change themselves [94]. Moreover, healthcare providers preferred a reflective tone instead of dictatorial to feedback [18, 28]. Our QCA found that both dictatorial and reflective interventions can be important in different situations. When all other important intervention components are present (training, dissemination of CS indications, multidisciplinary collaboration, willingness to change), dictated nature of feedback was not needed to evoke change, and thus reflective feedback was more beneficial. However, when other interventions were missing, dictated nature of interventions was important, possibly to strengthen the intervention delivery. Therefore, the decision about dictatorial or reflective intervention will depend on the structure of the other intervention components.

Lastly, use of theory in intervention design was important to maximise benefits and change behaviours [25, 92]. However, very few intervention studies aiming to optimise use of CS used implementation science or theory in intervention design, which represents a major limitation as evidence consistently demonstrates that the use of theoretically informed interventions leads to better outcomes [95, 96]. Future intervention studies addressing high rates of CS should use theory-based intervention design to ensure the potential mechanisms of action that may affect behaviour are adequately identified and targeted by the intervention, and that proposed interventions are known and able to influence the targeted behaviours [90, 97].

### Strength and limitations

Due to limited studies and imbalance in the number of successful and unsuccessful interventions, we cannot assess important components that may trigger success on implementation of financial reform, opinion leaders, mandatory opinion, and vaginal birth after caesarean (VBAC) policy. We also encountered challenges on detailed reporting of complex interventions (including implementation evaluation outcomes) which prevented us from engaging further with the interventions and may have missed important conditions that the studies have yet not reported*.* We have tried to compensate for this lack of detailed reporting through sibling studies search. Our QCA also did not contemplate endemic difference between high versus low- and middle-income countries (e.g. health systems functioning) that could explain the need of some conditions in certain countries but not in others. The Case:Condition ratio for the consolidated model could be a limitation of this analysis; to address this, we have run sub-analysis by adding more cases to ascertain that similar intermediate solutions are observed in a more balanced ratio. Lastly, we were unable to understand the impact of the interventions to changes on intrapartum versus elective CS, spontaneous vaginal birth, instrumental birth, or VBAC as studies did not consistently report these outcomes.

Our study is the first global analysis exploring how certain intervention components can influence the success of interventions targeting healthcare providers in the context of CS. This study used new analytical frameworks and existing evidence to generate new knowledge. The views synthesis and logical framework were used to ensure that the results are theory-driven and aligned with participants’ perspectives. Sensitivity analysis was also conducted to ensure the robustness of the study. Importantly, this study also extends the understanding of Chen et al. [19] CS intervention effectiveness review study by explaining potential intervention components which may influence heterogeneity. A critical strength of this QCA is that audit and feedback is one of the most common and accessible interventions; it is highly implemented and used and comprises a wide range of components that are normally considered in facilities. Thus, this analysis will be useful as a guide to increase success and optimise benefits when implementing one of the most prevalent intervention used to reduce unnecessary CS and used to increase quality of care and evidence-based practices in general.

#### Implications for practice, policy, and research

**Fig. 4:**
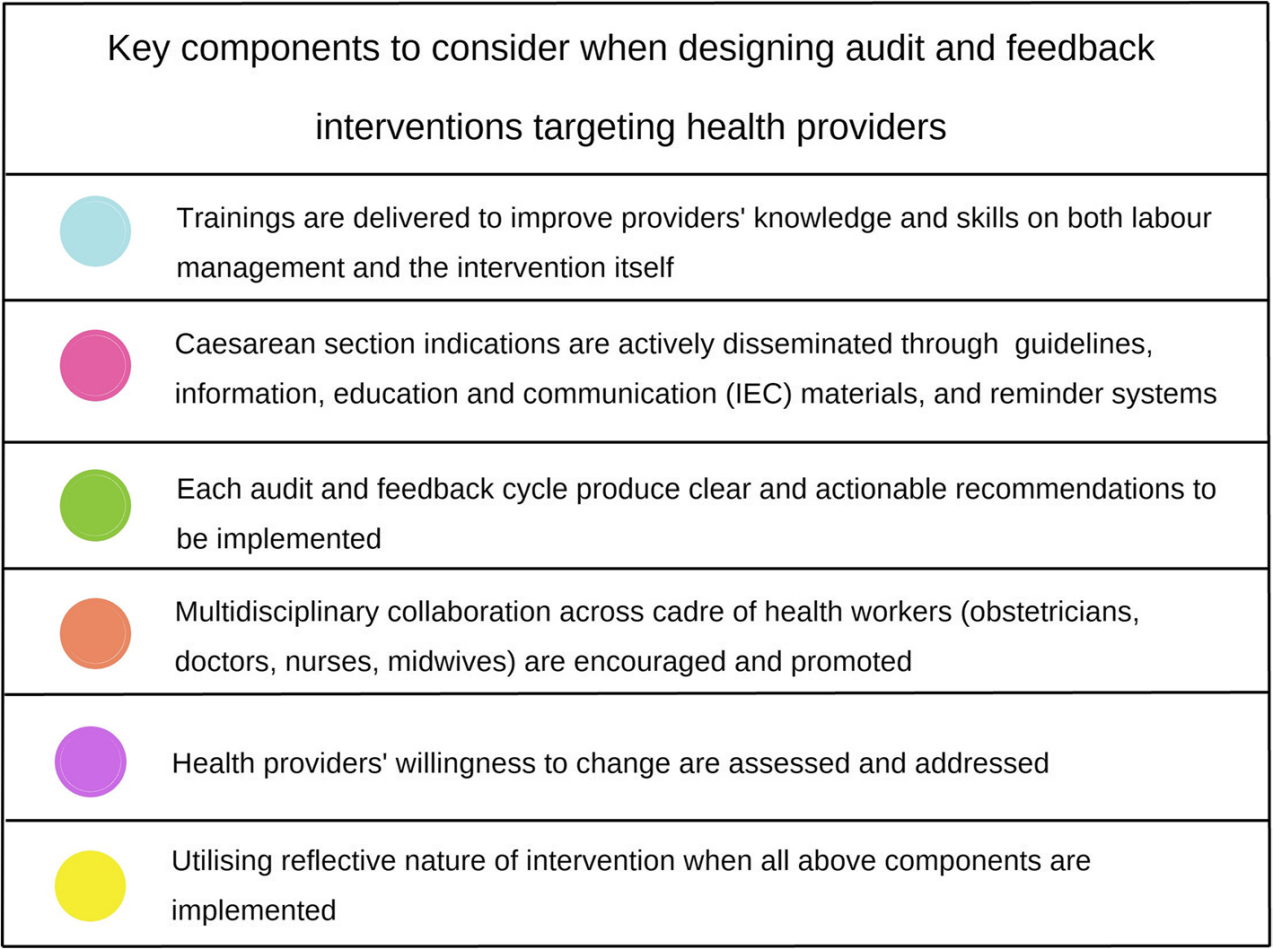
Checklist of key components to consider when designing interventions targeting healthcare providers. This figure can be used by healthcare providers, health facility managers, researchers, and policymakers as a checklist when designing interventions targeting healthcare providers to optimise CS use

When designing audit and feedback interventions targeting healthcare providers in the context of optimising CS, we recommend researchers, healthcare providers and institutions to consider the following key questions that may help lead to successful implementation, which are derived from our QCA findings (Fig. 4):

- 1. Are trainings to improve providers’ knowledge and skills on both the intervention and labour management implemented?
- 2. Are materials on CS indications actively disseminated to healthcare providers?
- 3. To what extent are providers willing to change behaviours regarding CS? Have their views been assessed and addressed (e.g. as part of formative research contributing to intervention design)?
- 4. Do audit and feedback cycles produce clear and actionable implementation recommendations?
- 5. Is multidisciplinary collaboration between obstetricians and midwives promoted when delivering care to women?
- 6. Based on questions 1–5, are dictated or reflective nature of interventions more appropriate?

Our QCA highlights the key role of the combination of provider training, active education and dissemination of CS indications, and audit and feedback which emphasize the need for adopting robust approaches to monitoring CS rates and indications at the health facility level. Although assessing and comparing CS rates and indications has been historically challenging and one of the barriers to implement effective measures to optimize the use of CS, in 2015, WHO recommended the use of the ten-group Robson classification. This system allows for a more standardized assessment and reporting of CS use and when adopted on regular basis, it assists to define concrete actions to improve practices [98, 99]. In 2021, WHO launched an interactive Platform for health facilities worldwide to share their data using the Robson classification and have data-driven conversations [100]. To increase to quality of evidence, more research is needed about interventions implementing financial reform, opinion leaders, mandatory second opinion, VBAC, multi-faceted, and multi-target interventions (targeting both women and healthcare providers). Future CS intervention studies should also ensure a complete reporting of intervention components implemented, including process outcomes, such as fidelity, attrition, adherence, contextual factors (details on what, where, when, how the interventions were delivered) and stakeholder perceptions of the interventions. This information is crucial in assessing not just if the intervention is successful, but also how similar interventions can work in one context and but not another.

## Conclusions

Our study is the first study that has investigated important intervention components and potential pathways which may trigger successful audit and feedback interventions targeting healthcare providers in the context of optimising CS use. Through our robust QCA, we identified five important components that jointly work together to promote successful outcomes 1) training to improve providers’ knowledge and skills, 2) active dissemination of CS indications, 3) actionable recommendations, 4) multidisciplinary collaboration, and 5) providers’ willingness to change. When designing the interventions targeting healthcare providers, health facility managers, researchers, and policymakers can consider the inclusion of the components above to ensure benefits. We also note that more research is needed on financial reform, opinion leaders, mandatory second opinion, VBAC, multi-faceted, and multi-target interventions (targeting both women and healthcare providers) and that study reports should include a detailed intervention process to ensure feasibility in examining heterogeneity in the future.

## Supplementary Information

**Additional file 1.** Logic model in optimizing CS use.

**Additional file 2.** Risk of bias assessment.

**Additional file 3.** Coding framework and calibration rules.

**Additional file 4.** Data table on the final models presented.

**Additional file 5.** Characteristics of included intervention studies.

**Additional file 6.** Data extracted from included studies.

## Data Availability

Data extracted and analysed on this study are available on Additional File 6. Any questions related to this can be sent to corresponding author: r.zahroh@unimelb.edu.au.

## Abbreviations

CovS: Coverage score
CS: Caesarean section
csQCA: Crisp set qualitative comparative analysis
fsQCA: Fuzzy set qualitative comparative analysis
IEC: Information, education, and communication
InclS: Inclusion score
LMICs: Low- and middle- income countries
PRI: Proportional reduction in inconsistency
QCA: Qualitative comparative analysis
VBAC: Vaginal birth after previous caesarean section
WHO: World Health Organization
