# Supplementary figures and images for: Interventions targeting healthcare providers to optimise use of caesarean section: a qualitative comparative analysis to identify important intervention features
Source: BMC Health Serv Res. 2022 Dec 14;22:1526. doi: 10.1186/s12913-022-08783-9 (PMC9753390; doi:10.1186/s12913-022-08783-9)

Additional file 1 – Logic model in optimizing CS use

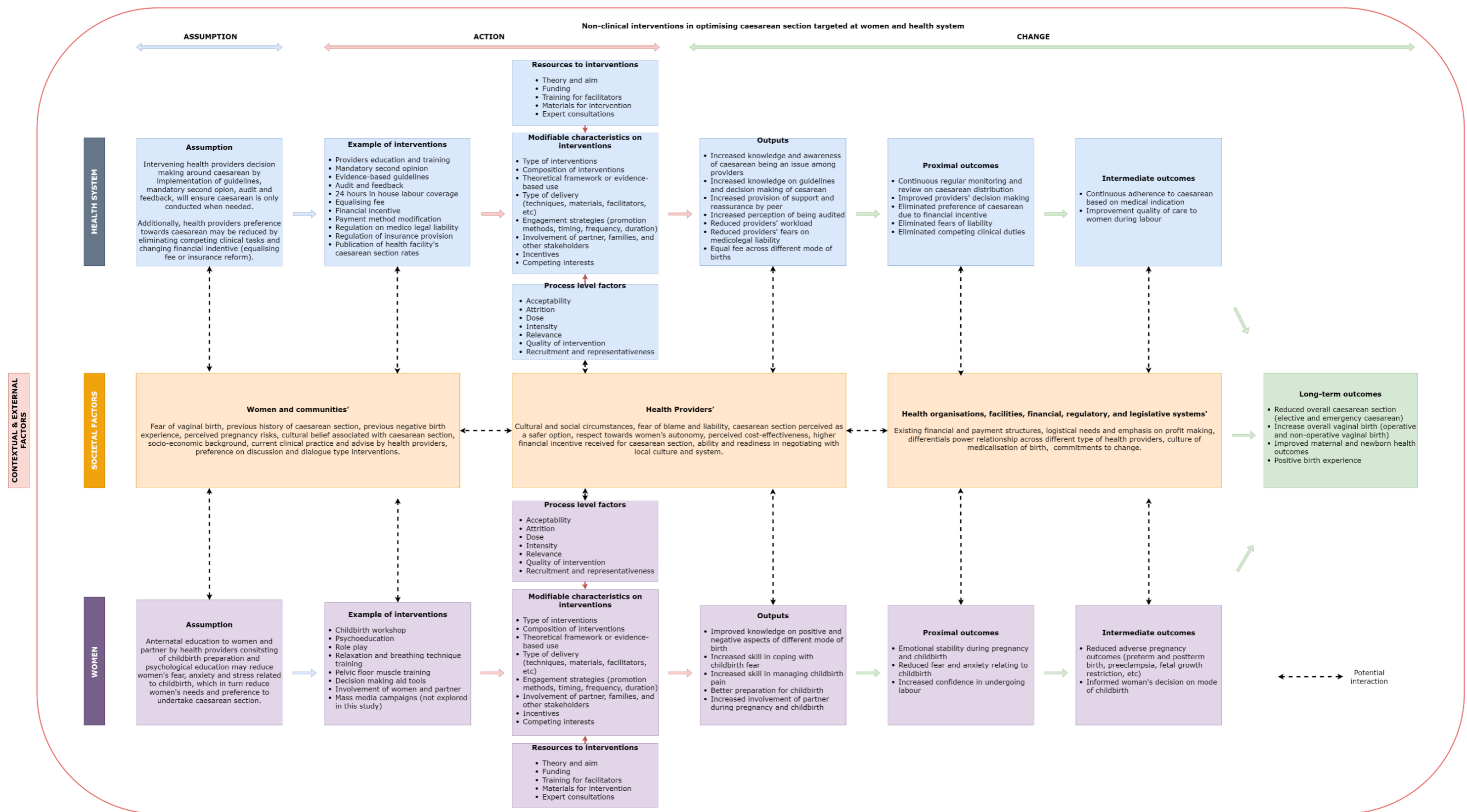

Supplement: Supplementary file 1 — Additional file 1. Logic model in optimizing CS use. [file 12913_2022_8783_MOESM1_ESM.pdf]
