# Supplementary material for: Interventions targeting healthcare providers to optimise use of caesarean section: a qualitative comparative analysis to identify important intervention features
Source: BMC Health Serv Res. 2022 Dec 14;22:1526. doi: 10.1186/s12913-022-08783-9 (PMC9753390; doi:10.1186/s12913-022-08783-9)
Supplement: Supplementary file 2 — Additional file 2. Risk of bias assessment. [file 12913_2022_8783_MOESM2_ESM.pdf]

Additional file 2 – Risk of bias assessment – overview

Author	Methods	Risks of Bias	Source	Tools
**Targeting healthcare providers or systems**				
**Calvo 2009**	Pre- and post-intervention design	Some concerns	Current study	Cochrane EPOC 'Risk of bias' criteria
**Ho 2011**	Before and after study	Some concerns	Current study	Cochrane EPOC 'Risk of bias' criteria
**Liang 2004**	Interrupted time series	Some concerns	Chen et al (2018)	Cochrane EPOC 'Risk of bias' criteria
**Mohammadi 2012**	Retrospective, before-after study	Some concerns	Chen et al (2018)	Cochrane EPOC 'Risk of bias' criteria
**Poma 1998**	Interrupted time series	Some concerns	Chen et al (2018)	Cochrane EPOC 'Risk of bias' criteria
**Scarella 2011**	Interrupted time series	Some concerns	Chen et al (2018)	Cochrane EPOC 'Risk of bias' criteria
**Bhartia 2020**	Interrupted time series	Some concerns	Current study	Cochrane EPOC 'Risk of bias' criteria
**Chaillet 2015**	Randomized controlled trial	Low risks of bias	Chen et al (2018)	Cochrane EPOC 'Risk of bias' criteria
**Kabore 2019**	Randomized controlled trial	Some concerns	Current study	Cochrane Risk of Bias Tool for randomised trials
**Kazandjian 1998**	Retrospective cohort, observational study	Satisfactory study	Current study	New-castle Ottawa Scale
**Lagrew 1996**	Interrupted time series	Some concerns	Current study	Cochrane EPOC 'Risk of bias' criteria
**Lomas 1991**	Randomized controlled trial	Some concerns	Chen et al (2018)	Cochrane EPOC 'Risk of bias' criteria
**Robson 1996**	Interrupted time series	Some concerns	Current study	Cochrane EPOC 'Risk of bias' criteria
**Socol 1993**	Interrupted time series	Some concerns	Current study	Cochrane EPOC 'Risk of bias' criteria
**van Dillen 2008**	Uncontrolled before-after study	Some concerns	Current study	Cochrane EPOC 'Risk of bias' criteria
**Multi-target interventions**				
**Xia 2019**	Uncontrolled before-after study	Low risks of bias	Current study	Cochrane EPOC 'Risk of bias' criteria
**Zhang 2020**	Randomized controlled trial	Some concerns	Current study	Cochrane Risk of Bias Tool for randomised trials
**Yu 2017**	Pre-post intervention study	Not serious	Opiyo et al (2020)	GRADE
**Borem 2020**	Interrupted time series	Not serious	Opiyo et al (2020)	GRADE

**Runmei 2012**	Controlled before-after study	Some concerns	Chen et al (2018)	Cochrane EPOC 'Risk of bias' criteria
**Clarke 2020**	Randomized controlled trial	Some concerns	Current study	Cochrane Risk of Bias Tool for randomised trials

Additional File 2.1 – Risk of bias assessment of observational study using Newcastle Ottawa Scale

**Domain**		**Kazandjian 1998**	
		**Score**	**Notes**
**Selection (Max. 5)**	**Representativeness**	1	Truly representative (all births during period of interest in the entire hospital network)
	**Sample size**	1	Justified and satisfactory
	**Non-respondents**	0	No provided
	**Measurement (exposure)**	1	Non-validated tool but described
**Comparibility (Max. 2)**	**Confounding control**	0	Non-participating hospitals as control
**Outcomes Measures & Analysis (Max. 3)**	**Assessment (outcome)**	1	Unblinded
	**Statistical test**	1	Statistical test used and appropriate
**Final Score / Final Assessment**		5	Satisfactory study

Additional File 2.2 – Risk of bias assessment of randomised controlled trial using Cochrane Risk of Bias Tool for randomised trials

**Author**	**Kabore 2019**	**Clarke 2020**	**Zhang 2020**
**Risk of bias arising from the randomization process**	Some concerns	Some concerns	Low risks
**Notes**	Allocation random and concealed after assignment. Higher previous CS history in intervention group	Allocation random and concealed after assignment. Significantly higher number of women in the intervention group with	Allocation random and concealed after assignment. No significant baseline differences

		higher number of VB history (before previous births)	
**Risk of bias arising from the timing of identification or recruitment of participants**	Low risks	Low risks	Low risks
**Notes**	Cluster (hospitals) recruited before randomisation but women were recruited after randomisation	Cluster (hospitals) recruited before randomisation but women were recruited after randomisation	Hospitals are recruited before randomisation
**Risk of bias due to deviations from the intended interventions**	Low risks	Some concerns	Low risks
**Notes**	Participants (women and healthcare providers) are aware of the trials and may be aware of the assigned intervention	Participants (women and healthcare providers) are aware of the trials and may be aware of the assigned intervention. It was mentioned that "the trial team were blinded to the results of the trial" but not the the assigned intervention	Participants (women and healthcare providers) are aware of the trials and may be aware of the assigned intervention. It was mentioned that "No masking was applied in this study"
**Risk of bias due to missing outcome data**	Low risks	Low risks	Low risks
**Notes**	No missing outcome data	No missing outcome data	Random selection at pre- and post-intervention
**Risk of bias in measurement of the outcome**	Low risks	Some concerns	Some concerns
**Notes**	Seems that the outcomes assessor is blinded -> "Data collectors were aware of the randomization assignments but were not involved in outcome assessments. Access to the database was restricted to the data manager until the trial was completed"	Unclear if the outcomes assessor is blinded or not	Unclear if the outcomes assessor is blinded or not

**Risk of bias in selection of the reported result**	Low risks	Low risks	Low risks
**Notes**	Seems to be intention to trear analysis. Results are not assessed based on multiple measurements or multiple analysis	Seems to be intention to treat analysis. Results are not assessed based on multiple measurements or multiple analysis	Seems to be intention to trear analysis. Results are not assessed based on multiple measurements or multiple analysis
**Final Assesment**	Some concerns	Some concerns	Some concerns

Additional File 2.3 – Risk of bias assessment of uncontrolled before and after study using Cochrane EPOC 'Risk of bias' criteria

Author	Intervention independent of other changes	Notes	Shape of the intervention effect pre-specified	Notes	Intervention unlikely to affect data collection	Notes	Knowledge of the allocated interventions adequately prevented during the study	Notes	Incomplete outcome data (attrition bias)	Notes	Selective outcome reporting (reporting bias)	Notes	Other risks of bias	Notes	Final Assesment
**Calvo 2009**	Unclear risks	Unclear, not stated	Low risks	Point of intervention and analysis seems relevant	Low risks	Sources and methods of data collection were the same before and after the intervention: using medical records	Low risks	Outcomes are objectively assessed	Unclear risks	Unclear how many missing data	Low risks	All relevant outcomes in the methods section are reported in the results section	N/A	N/A	Some concerns
**Ho 2011**	Low risks	Covariance assessed	Low risks	Point of intervention and analysis seems relevant	Low risks	Sources and methods of data collection were the same before and after	Low risks	Outcomes are objectively assessed	Unclear risks	Unclear how many missing data	Low risks	All relevant outcomes in the methods section	N/A	N/A	Some concerns

						the intervention: using medical records						are reported in the results section			
**Bhartia 2020**	Low risks	Contributing factors are related to the implemented interventions	Low risks	Point of intervention and analysis seems relevant	Low risks	Sources and methods of data collection were the same before and after the intervention: using medical records	Low risks	Outcomes are objectively assessed	Unclear risks	Unclear how many missing data	Low risks	All relevant outcomes in the methods section are reported in the results section	N/A	N/A	Some concerns
**Lagrew 1996**	Unclear risks	Unclear, not stated	Low risks	Point of intervention and analysis seems relevant	Low risks	Sources and methods of data collection were the same before and after the intervention: using medical records	Low risks	Outcomes are objectively assessed	Unclear risks	Unclear how many missing data	Low risks	All relevant outcomes in the methods section are reported in the results section	Some concerns	Unclear how long is the intervention implementation	Some concerns

**Robson 1996**	Some concerns	There could have been other historic events during this period that weren't mentioned	Low risks	Point of intervention and analysis seems relevant	Low risks	Sources and methods of data collection were the same before and after the intervention: using medical records	Low risks	Outcomes are objectively assessed	Unclear risks	Unclear how many missing data	Low risks	All relevant outcomes in the methods section are reported in the results section	Some concerns	Unclear intensity of the audit and feedback during 1989-1992 (prospective/retrospective audit?).	Some concerns
**Socol 1993**	Unclear risks	Unclear, not stated	Low risks	Point of intervention and analysis seems relevant	Low risks	Sources and methods of data collection were the same before and after the intervention: using medical records	Low risks	Outcomes are objectively assessed	Unclear risks	Unclear how many missing data	Low risks	All relevant outcomes in the methods section are reported in the results section	N/A	N/A	Some concerns
**van Dillen 2008**	Unclear risks	Unclear, not stated	Low risks	Point of intervention and analysis seems relevant	Low risks	Sources and methods of data collection were the	Low risks	Outcomes are objectively assessed	Low risks	3% missing data	Low risks	All relevant outcomes in the	N/A	N/A	Some concerns

						same before and after the intervention: using medical records						methods section are reported in the results section			
**Xia 2019**	Low risks	Covariance assessed	Low risks	Point of intervention and analysis seems relevant	Low risks	Sources and methods of data collection were the same before and after the intervention: using medical records	Low risks	Outcomes are objectively assessed	Low risks	1000 out of almost 2,000,000 data	Low risks	All relevant outcomes in the methods section are reported in the results section	N/A	N/A	Low risks of bias
