# Supplementary material for: Interventions targeting healthcare providers to optimise use of caesarean section: a qualitative comparative analysis to identify important intervention features
Source: BMC Health Serv Res. 2022 Dec 14;22:1526. doi: 10.1186/s12913-022-08783-9 (PMC9753390; doi:10.1186/s12913-022-08783-9)
Supplement: Supplementary file 3 — Additional file 3. Coding framework and calibration rules. [file 12913_2022_8783_MOESM3_ESM.pdf]

Additional File 3 – Coding framework and calibration rules

Condition	Field	Instructions for extractors	Calibration type	Coding values and calibration rules
**Domain 1**	**Setting and participants (7 conditions)**			
**1.1**	Number of participants	Recorded total number of participants involved in intervention	Not calibrated	All studies did not report number of providers but instead only number of women exposed to the interventions, except one study (37).
**1.2**	Composition of participants	What were the composition of participants? Is it only women, or women and partners?	Direct assignment	Healthcare providers: = yes: 1, no: 0<br>Engaging women: = yes: 1, no: 0
**1.3**	Intervention location	Did the interventions delivered at health facilities, home, or communities?	Direct assignment	Health facility = yes: 1, no: 0
**1.4**	Number of health facility	Recorded number of health facilities implementing the intervention	Transformational assignment (Ragin's direct method)	100 or more health facilities = 0<br>10 or more health facilities = 0.5<br>10 or fewer = 1<br><br>Other values span between 0 to 1
**1.5**	Health facility type	Was the health facility public or private owned?	Direct assignment	Public: = yes: 1, no: 0<br>Private = yes: 1, no: 0
**1.6**	Baseline CS rates	Recorded baseline CS rates at the health facility	Direct assignment	High baseline CS rates (>15%) at study facility = 1<br>High baseline CS rates (>15%) at area/country= 0.66<br>Not reported = 0<br>Not considering baseline rates but mention high preference of CS/increasing CS = 0.33<br>Not considering CS at all OR low based CS rates<10% CS rates = 0
**Domain 2**	**Intervention design (24 conditions)**			
**2.1**	Component of interventions	Extract the type of interventions included: i.e antenatal education, psychosocial education, audit and feedback implementation	Direct assignment to all listed interventions	Audit and feedback: yes = 1, no = 0<br>Training for healthcare providers: yes = 1, no = 0<br>Actionable recommendations of each audit and feedback cycle: yes = 1, no = 0<br>Multi-disciplinary collaboration: yes = 1, no = 0

				Organisation of care, including 24-h labour coverage: yes = 1, no = 0<br>Mandatory second opinion: yes = 1, no = 0<br>Opinion leaders: yes = 1, no = 0<br>VBAC advocacy: yes = 1, no = 0<br>Incentives as reward system: yes = 1, no = 0<br>Robson implementation: yes = 1, no = 0<br>Improvement collaboration participation: yes = 1, no = 0<br>Active dissemination of CS indications: yes = 1, no = 0
**2.2**	Utilisation of theoretical framework or evidence-based intervention	Did the study name a theoretical framework that underpins the intervention design or delivery style?	Direct assignment	Theory driven = 1<br>Citing previous study only = 0.33<br>Missing/Not using any/ambiguous = 0
**2.3**	Type of intervention facilitators or instructors	Who were the instructors or facilitators of the intervention? i.e. peers, obstetrician, midwives, doula, other trained staff	Direct assignment to all listed facilitators	Maternity healthcare providers (nurse, midwife, obstetrician): yes = 1; not mentioned as an instructor/facilitator = 0<br>Hospital administrators: yes = 1; not mentioned as an instructor/facilitator = 0<br>Recruited staffs: yes = 1; not mentioned as an instructor/facilitator = 0
**2.4**	Personal or group delivery (training)	Did the intervention delivered in a group or personal based delivery through workshop or class?	Direct assignment	Group delivery: yes = 1, no = 0<br>Personalised/individualised: yes = 1, no = 0
**2.5**	Audit and feedback results dissemination	Did audit and feedback delivered in public or one to one?	Direct assignment	Publicly: yes = 1, no = 0<br>One to one: yes = 1, no = 0
**2.6**	Language tone of the intervention	Is language tone one the interventions reflective or dictated?	Direct assignment	Reflective: yes = 1, no = 0<br>Dictated: yes = 1, no = 0

**2.7**	Type of materials used	Did the intervention used specific type of materials? i.e. video, computer-based tool, pamphlet, handbook, guidelines	Direct assignment to all listed materials	IEC materials: yes = 1, no/not reported = 0<br>Guidelines dissemination: yes = 1, no/not reported = 0
**2.8**	Information about control condition	Described whether the authors provided a control for the main intervention (intended to capture complexity of running an intervention and a control) - are they equivalent or not?	Direct assignment	No control described/ambiguous = 0<br>Yes, there is control group = 1
**Domain 3**	**Program content (6 conditions)**			
**3.1**	Content delivered to healthcare providers	What type of content themes or curriculum delivered to mother and partners? i.e. relaxation technique, information on vaginal birth vs caesarean section risks	Direct assignment to all listed content	CS indication: yes = 1, no = 0<br>Labour management: yes = 1, no = 0<br>VBAC: yes = 1, no = 0<br>Women's autonomy: yes = 1, no = 0
**3.2**	Educational or training technique	What were the education or training technique used? i.e. role-play, lecture-based, study cases	Direct assignment to all listed content	Practice based (practical session, behavioural rehearsal, role play): yes = 1, no = 0, not reported = 0<br>Lecture/didactic based: yes = 1, no = 0, not reported = 0
**Domain 4**	**Engagement (3 conditions)**			
**4.1**	Involvement with partners, families, other stakeholders	Other than participants, were there any other stakeholders included in the intervention?	Direct assignment	Yes = 1<br>No = 0
**4.2**	Frequency of engagement	How often did the intervention delivered (or frequency of engagement/encounter between participants and intervention)? i.e. once in a month, every 3 months	Direct assignment	Weekly to monthly = 1<br>Every two or three months = 0.75<br>Not reported/Annual = 0

**4.3**	Existing competing interest	Was there any competing interest stated?	Direct assignment	No = 1<br>Yes/ Not reported = 0
**Domain 5**	**Health system (12 conditions)**			
**5.1**	Presence of internal policies	Were there any existing policies in place to support the intervention (both at facility, regional, or country level)?	Direct assignment	No = 0<br>Not reported = 0<br>Yes = 1
**5.2**	Presence of any other support and resources	Were there any existing support from health system which directly or indirectly support the intervention?	Direct assignment	No = 0<br>Not reported = 0<br>Yes = 1
**5.3**	Perceptions of stakeholders related to the interventions or the caesarean section itself	What were the perceptions of both participants and other relevant stakeholders in regard to caesarean section?	Direct assignment	Missing/not reported = 0<br>Negative = 0.33<br>Medium/neutral = 0.5<br>More positive, despite few dissatisfaction = 0.66<br>Positive = 1
**5.4**	Local adaptation	Any acknowledgment to local adaptation?	Direct assignment	No = 0<br>Not reported = 0<br>Yes = 1
**5.5**	Willingness to change	Do providers show willingness in engaging with interventions or change on CS practice?	Direct assignment	No = 0<br>Not reported = 0<br>Yes = 1
**5.6**	Obstacles perceived	Were there any obstacles mentioned by participants or trialists?	Direct assignment	Complexity of systems: yes = 1, no = 0, not reported = 0<br>Perceived poor training, skills and experience: yes = 1, no = 0, not reported = 0<br>Unsupportive hierarchies/poor teamwork: yes = 1, no = 0, not reported = 0<br>Fear of blame: yes = 1, no = 0, not reported = 0
**5.7**	Belief associated with CS	Recorded any belief regarding CS and birth from providers' perspective	Direct assignment	Belief on unnecessary CS: yes = 1, no = 0, not reported = 0<br>Belief on women's autonomy: yes = 1, no = 0, not reported = 0

				Norms on CS as acceptable: yes = 0, no = 1, not reported = 0
**Domain 6**	**Process outcomes (5 conditions)**			
**6.1**	Intervention fidelity	Extract level of fidelity (%) or extract qualitative statement on intervention fidelity. Intervention fidelity refers to the degree to which the prescribed components of the intervention, as described in the study protocol, have been delivered	Direct assignment	There is evidence on high fidelity = 1<br>Med fidelity 0.66<br>Low fidelity = 0.33<br>Not reported = 0
**6.2**	Participants satisfaction	Extract level of satisfaction (%) or extract qualitative statement on participants' satisfaction with the intervention experience	Direct assignment	Direct assignment - qualitative: where there is a positive statement = 0.66; negative statement = 0.33; where not mentioned = 0<br><br>Direct assignment - quantitative: Interventions with 25% or fewer participants satisfied = 0; with 50% of participants satisfied = 0.5; not mentioned = 0; interventions with >75% participants satisfied = 1
**6.3**	Participants attrition	Extract participants' level of completion (%) or record qualitative statement on participants' completion rate	Direct assignment	Direct assignment - qualitative: where there is a statement indicating high level of completion, assign value of 0.66; where a statement indicating problematic completion, assign value of 0.33. Where data are missing, assign value of 0<br><br>Direct assignment - quantitative: Interventions with 66% or fewer participants completing the intervention = 0; interventions with 75% of participants completing the intervention = 0.5; interventions with 83% or more participants completing the intervention = 1. Missing data coded as 0

**6.4**	Participants dosage level	Did the participant receive the intended dosage of the intervention? "intended dosage is the amount of the components of the intervention delivered by implementers and the extent to which participants received and used materials or other resources". Extract level of dosage (%) or record qualitative statement	Direct assignment	Direct assignment - qualitative: where there is a qualitative statement indicating high level of dosage, assign value of 0.66; where a qualitative statement indicating problematic dosage, assign value of 0.33. Where data are missing, assign value of 0<br><br>Direct assignment - quantitative: Interventions with 66% or fewer participants receiving the full dosage = 0; interventions with 75% of participants receiving the full dosage = 0.5; interventions with 83% or more of participants receiving the full dosage = 1. Missing data coded as 0
**6.5**	Participant adherence	Did participants adhere to the intervention instructions, e.g. women and partners attending classes; healthcare providers conducted mandatory second opinion. Extract level of adherence (%) or qualitative statement		Direct assignment - qualitative: where there is a statement indicating high level of adherence, assign value of 0.66; where a qualitative statement indicating problematic adherence, assign value of 0.33. Where data are missing, assign value of 0<br><br>Direct assignment - quantitative: Interventions with 66% or fewer participants adherent = 0; interventions with 75% of participants adherent = 0.5; interventions with 83% or more participants adherent = 1. Missing data coded as 0
**6.6**	Collateral effect other than to participants	Is there any stated collateral effects on others (other than participants)? i.e. if women obtain 24/7 epidural but no additional human resources are provided to the unit, doctors may need to do more shifts or work longer hours to their in satisfaction.	Not coded	Not coded only for consideration whether the intervention is successful or not

**6.7**	Any other factors affecting the success/unsuccessful of the implementation?	Any other factors affecting the success/unsuccessful of the implementation?	Not coded	Not coded only for consideration whether the intervention is successful or not
**Domain 7**	**Type of interventions (1 condition)**			
**7.1**	Type of interventions	What is the type of interventions? Is it interventions targeting women or multi-target interventions?	Direct assignment	Multi-target interventions: yes = 1, no = 0
