# Supplementary material for: Interventions targeting healthcare providers to optimise use of caesarean section: a qualitative comparative analysis to identify important intervention features
Source: BMC Health Serv Res. 2022 Dec 14;22:1526. doi: 10.1186/s12913-022-08783-9 (PMC9753390; doi:10.1186/s12913-022-08783-9)
Supplement: Supplementary file 4 — Additional file 4. Data table on the final models presented. [file 12913_2022_8783_MOESM4_ESM.pdf]

Additional File 4 – Data table on the final models presented

Author	CS outcome	Frequent engagement	Actionable recommendations	Active dissemination of CS indications	Multi-disciplinary collaboration	Providers' willingness to change	Internal policies	Engaging women	Dictated	Training to improve knowledge and skills	Individual dissemination	Multi-target intervention
**Interventions targeting healthcare providers**												
**Calvo 2009**	0	1	1	1	0	0	0	0	1	1	0	0
**EC9 Ho 2011**	0	0	0	0	1	0	1	0	0	1	0	0
**Liang 2004**	0	1	1	1	0	0	1	0	1	1	0	0
**Mohammadi 2012**	0	1	0	1	0	0	0	0	1	0	1	0
**Poma 1998**	0	0	0	1	0	0	1	0	0	0	1	0
**Scarella 2011**	0	1	0	1	1	0	1	0	0	0	1	0
**Bhartia 2020**	1	1	1	0	1	1	1	0	0	1	0	0
**Chaillet 2015**	1	1	1	1	1	0	0	0	0	1	0	0
**Kabore 2019**	1	1	1	1	1	0	0	0	0	1	1	0
**Kazandjian 1998**	1	0	1	0	1	0	0	0	1	1	0	0
**Lagrew 1996**	1	1	1	0	0	1	0	1	0	1	1	0
**Lomas 1991**	1	1	1	1	0	1	0	1	0	0	0	0
**Robson 1996**	1	1	1	0	1	1	0	1	0	1	1	0
**Socol 1993**	1	0	1	1	0	1	1	0	0	0	1	0

**van Dillen 2008**	1	1	1	1	1	1	0	0	0	0	0	0
**Multi-target interventions**												
**Zhang 2020**	0	0	1	0	0	0	0	1	1	1	0	1
**Borem 2020**	1	1	1	0	1	0	0	1	1	1	0	1
**Clarke 2020**	1	1	1	0	1	0	1	1	1	1	0	1
**Runmei 2012**	1	0	1	1	0	0	0	1	1	1	0	1
**Xia 2019**	1	0	1	0	1	0	1	1	1	1	0	1
**Yu 2017**	1	1	1	1	0	0	1	1	1	1	0	1
