# Supplementary material for: Interventions targeting healthcare providers to optimise use of caesarean section: a qualitative comparative analysis to identify important intervention features
Source: BMC Health Serv Res. 2022 Dec 14;22:1526. doi: 10.1186/s12913-022-08783-9 (PMC9753390; doi:10.1186/s12913-022-08783-9)
Supplement: Supplementary file 5 — Additional file 5. Characteristics of included intervention studies. [file 12913_2022_8783_MOESM5_ESM.pdf]

Additional File 5. Characteristics of included intervention studies

Author	Sibling studies	Country & study years	Intervention	Methods	Settings	Type of women	Sample	Baseline CS rates	Relative effect	CS Outcomes	Risk of Bias
**Interventions targeting healthcare providers**											
**Calvo 2009**	None	Spain 2006-2007	Audit and feedback, protocol implementation, weekly debate, courses for providers and the correction of deficiencies in organization (structural and organizational; not clearly mentioned)	Before and after study	Two public Spanish hospitals	All pregnant women	Unclear	17.5% and 29.0%	Overall CS rates at decreased (not statistically significant) from 17.5 to 15.8% and from 29.0 to 22.0%, respectively	Little or no difference	Some concerns
**Ho 2011**	2 studies: protocol (56) and methodology (development of logical framework) (57)	Malaysia 2005-2008	Audit and feedback, opinion leader's appointment, evidence-based workshops for nurses and doctors, training, academic exchanges	Before and after study	Two Malaysian hospital	All pregnant women	4628	43.0%	Unadjusted RD 7.75 (95% CI 5.3 to 10.2) and adjusted RD 7.56 (2.92 to 12.20)	Increase	Some concerns
**Liang 2004**	None	Taiwan 1997-2000	Audit and feedback, mandatory second opinion, weekly conference, trial of labour after one CS	Interrupted time series	A tertiary hospital	All pregnant women indicated with CS	35,602	37.0%	Change in level of total CS at 12 months: -2.4% (-11.4 to 6.7) Change in slope: 1.34% (-2.5 to 5.2)	Uncertain	Some concerns

**Mohammadi 2012**	None	Iran 2005	Audit and feedback, financial incentives	Before and after study	An Iranian hospital	All pregnant women	3494	40.0%	Change in level of total CS at 12 months: -14.6% (-24.4 to -4.8), P = 0.02 Change in slope: -0.07% (-1.5 to 1.3)	Uncertain	Some concerns
**Poma 1998**	None	USA 1991-1996	Audit and feedback, guidelines implementation, review meetings, publication of anonymous individual and department rates, 24-hour in-house coverage	Interrupted time series	Community hospital obstetric unit	All pregnant women	12,912	22.5%	Change in level of total CS at 12 months: -6.6% (-10.1 to -3.2) Change in slope: -0.11% (-0.25 to 0.02)	Uncertain	Some concerns
**Scarella 2011**	None	Chile 2007-2008	Audit and feedback, Robson classification implementation, monthly CS rates distributed by letters, meetings every three month	Interrupted time series	A hospital (maternity ward)	All pregnant women admitted in spontaneous labor or for pregnancy interruption	4831	36.8%	Change in level of CS during intervention: -11% (-23.2 to 1.2), NS; change in slope -1.1% (-6.4 to 4.2)	Uncertain	Some concerns
**Bhartia 2020**	1 study: collaboration development (58)	India 2011-2016	Audit and feedback, antenatal class, additional providers recruitment, protocol/guidelines development and	Interrupted time series	A private hospital in India	All pregnant women	Unclear	79.0%	Between 2011 and 2016, the CS rate declined from 52 to 18%, (p < 0.001)	Decrease	Some concerns

			implementation, international improvement collaboration, regular training, empowerment of junior obstetrician, group practice, VBAC promotion								
**Chaillet 2015**	2 studies: budget impact analysis (59) and cost-effectiveness analysis (60)	Canada 2008-2011	Audit and feedback, providers' training, evidence-based guidelines, local opinion leader	Randomized controlled trial	32 hospitals in Quebec	All pregnant women	184,952	22.50%	RD −1.8 (−3.8 to −0.2)<br>OR 0.90 (0.80 to 0.99)	Decrease	Low risks of bias
**Kabore 2019**	2 studies: study protocol (61) and observational study (62)	Burkina Faso 2014-2016	Audit and feedback, clinical algorithms (protocol/guidelines), providers' training, dissemination of algorithms through posters, and weekly SMS reminders	Randomized controlled trial	22 referral hospitals	All pregnant women	4174	21.40%	The overall CS rate changed from 21.4% (baseline) to 20.0% (post-intervention) in the hospitals in the intervention group and from 18.7% (baseline) to 20.3% (post-intervention) in the control hospitals (absolute risk difference −	Decrease	Some concerns

									3.1%; 95% CI – 7.2 to 2.6%, P = 0.203)		
**Kazandjian 1998**	2 studies: conceptual framework development (55) and case studies (63)	USA 1991-1996	Adoption of reporting system on caesarean section (QI Project) - similar to audit and feedback	Observational study	Hospitals	All pregnant women	Unclear	22.5%	The total CS rate declined from 22.5% in 1991 to 19.4% in 1996 (p<0.01)	Decrease	Satisfactory study
**Lagrew 1996**	None	USA 1998-1994	Audit and feedback, guidelines implementation, education, program promotion, prenatal education on VBAC, departmental conferences and meetings	Interrupted time series	A private hospital	All pregnant women	12,118	31.1%	The overall CS rate fell from 31.1 % to 15.4% (p<0.000001)	Decrease	Some concerns
**Lomas 1991**	1 study: cross-sectional study (64)	Canada 1988-1989	Audit and feedback, local opinion leader, guidelines implementation	Randomized controlled trial	16 community hospitals	All pregnant women		18.3% and 20.2%	Control: 85.5%, Audit and feedback: 88.3%, opinion leader: 75.1%	Decrease	Some concerns
**Robson 1996**	None	USA 1984-1992	Medical audit cycle implementation	Interrupted time series	A hospital	All pregnant women	21,125	12.0%	The overall CS rate was decreased (9.5% vs 12%, p < 0.0001)	Decrease	Some concerns
**Socol 1993**	None	USA 1987-1991	Audit and feedback, guidelines implementation	Interrupted time series	A private tertiary university facility	All pregnant women	26,894	27.3%	The overall CS rate fell from 27.3% to 16.9% (p < 0.0001)	Decrease	Some concerns

**van Dillen 2008**	None	Netherlands 2005-2006	Audit and feedback, daily report meetings	Before and after study	A teaching hospital	All pregnant women	1221	23.4%	The overall CS rate fell from 23.4% to 18.7%	Decrease	Some concerns
**Multi-target interventions**											
**Zhang 2020**	None	China 2015 - 2017	Targeted health education to pregnant women, improved hospital CS policy, and training of midwives/doulas	Randomized controlled trial	Tertiary and secondary hospitals	Women with low-risk pregnancy	10,752	42.50% at facility level	OR = 0.92; 95% CI 0.73, 1.15	Little or no difference	Some concerns
**Borem 2020**	None	Brazil 2014 - 2016	A coalition of stakeholders, empowerment of pregnant women to choose mode of delivery, psychologic birth promotion, information system for providers	Interrupted time series	Hospitals	Women with low-risk pregnancy	119,378	78.3% at facility level	Vaginal deliveries RR 1.62 (95% CI 1.27 to 2.07, p < 0.001)	Decrease	Not serious
**Clarke 2020**	3 studies: process evaluation (65), protocol (66), formative qualitative (67)	Italy, Ireland, Germany 2012 - 2016	Education of clinicians and women with one previous CS, appointment of opinion leaders, audit/peer review, and joint discussions by women and clinicians	Randomized controlled trial	Maternity units	Women with previous CS	2,002	VBAC rates < 35% at facility level	RR 0.9 (95%CI 0.85 to 0.98)	Decrease	Some concerns
**Runmei 2012**	None	China 2005 - 2011	Education for staffs and women, audits, public health education, monitoring	Before and after study	Regional referral centre	Women with low-risk pregnancy	25,280	53.5%-56.1% at facility level	OR 0.56 (0.52–0.59)	Decrease	Some concerns

			CS rates, and neonatal outcomes								
**Yu 2017**	None	China 2006 - 2014	Face-to-face weekly educational meetings between patients and hospital staff, training for providers, new regulations adoption and projects on CS (i.e. encourage mothers to choose vaginal delivery, strictly control indications for CS and maternal request for CS)	Before and after study	Tertiary public hospitals	Women with low-risk pregnancy	131,312	55-56% at facility level	Overall CS rate declined by 1.29% (p = 0.002)	Decrease	Not serious
**Xia 2019**	None	China 2010 - 2016	Programs for population health education, skills training for healthcare professionals, equipment and technical support for local healthcare facilities, and capacity building for the maternal near-miss care system	Before and after study	Hospitals and community	Women with low-risk pregnancy	1,923,687	42% at facility level	Decreasing trend in the monthly CS rates (Z = 75.067, p < 0.001)	Decrease	Low risks of bias
