# Supplementary material for: Interventions targeting healthcare providers to optimise use of caesarean section: a qualitative comparative analysis to identify important intervention features
Source: BMC Health Serv Res. 2022 Dec 14;22:1526. doi: 10.1186/s12913-022-08783-9 (PMC9753390; doi:10.1186/s12913-022-08783-9)
Supplement: Supplementary file 6 — Additional file 6. Data extracted from included studies. [file 12913_2022_8783_MOESM6_ESM.pdf]

Additional File 6. Data extracted from included studies

Data extracted from studies and recoded is available at this link: https://bit.ly/3KAuB2U

Please contact the corresponding author with any requests or clarifications: Rana Islamiah Zahroh, r.zahroh@unimelb.edu.au
